# Electrical coupling regulates layer 1 interneuron microcircuit formation in the neocortex

**DOI:** 10.1038/ncomms12229

**Published:** 2016-08-11

**Authors:** Xing-Hua Yao, Min Wang, Xiang-Nan He, Fei He, Shu-Qing Zhang, Wenlian Lu, Zi-Long Qiu, Yong-Chun Yu

**Affiliations:** 1 Institute of Neurobiology, Institutes of Brain Science, State Key Laboratory of Medical Neurobiology and Collaborative Innovation Center for Brain Science, Fudan University, Shanghai 200032, China; 2 Centre for Computational Systems Biology and the School of Mathematical Sciences, Fudan University, Shanghai 200433, China; 3 Stem Cell Translational Research Center, Tongji Hospital, Tongji University School of Medicine, Shanghai 200092, China; 4 Institute of Neuroscience and State Key Laboratory of Neuroscience, Shanghai Institutes for Biological Sciences, Chinese Academy of Sciences and University of Chinese Academy of Sciences, 320 Yue-Yang Road, Shanghai 200031, China

## Abstract

The coexistence of electrical and chemical synapses among interneurons is essential for interneuron function in the neocortex. However, it remains largely unclear whether electrical coupling between interneurons influences chemical synapse formation and microcircuit assembly during development. Here, we show that electrical and GABAergic chemical connections robustly develop between interneurons in neocortical layer 1 over a similar time course. Electrical coupling promotes action potential generation and synchronous firing between layer 1 interneurons. Furthermore, electrically coupled interneurons exhibit strong GABA-A receptor-mediated synchronous synaptic activity. Disruption of electrical coupling leads to a loss of bidirectional, but not unidirectional, GABAergic connections. Moreover, a reduction in electrical coupling induces an increase in excitatory synaptic inputs to layer 1 interneurons. Together, these findings strongly suggest that electrical coupling between neocortical interneurons plays a critical role in regulating chemical synapse development and precise formation of circuits.

## Abstract (web summary)

Electrical and chemical synapses coexist in neocortex interneurons. Here, Yao *et al*. show that during development, electrical and GABAergic chemical synapses form at the same time, and that this coupling synchronizes firing between layer 1 interneurons.

## Graphical abstract

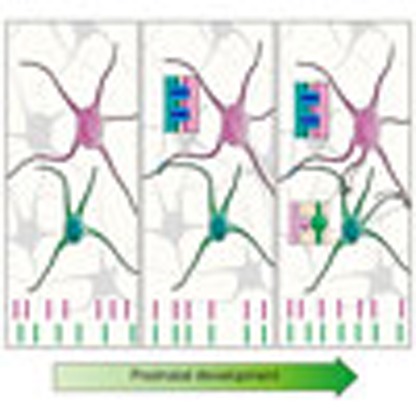

During brain development, gap junction-mediated cell coupling has been shown to play a critical role in processes, such as neurogenesis^1^,^2^,^3^,^4^, migration^5^,^6^, cellular differentiation^7^, circuit formation^8^,^9^ and synchronization^10^,^11^,^12^,^13^,^14^,^15^. It is generally believed that gap junction-mediated neuronal communication regulates the formation of chemical synapses^9^. For example, studies have shown that transient electrical synapses modulate the assembly of precise chemical synapses between sister excitatory neurons in neocortical ontogenetic columns^16^, and further influence orientation selectivity tuning^17^. While these studies provide crucial insights into the roles of gap junctions in the functional development and circuit assembly of excitatory neurons, whether electrical coupling modulates chemical synapse formation of interneurons remains largely unknown.

Accumulating evidence suggests that neocortical synaptogenesis in excitatory neurons and inhibitory interneurons are drastically different^10^,^16^,^18^,^19^,^20^. During the first postnatal week, when electrical synapses are abundantly present in excitatory neurons, chemical synapses are largely undetectable^20^. As electrical synapses approach the time point of their elimination, chemical connections between excitatory neurons begin to emerge, illustrating a sequential developmental time course for the two types of connections in excitatory neurons^16^,^19^,^20^. By contrast, chemical and electrical synapses between neocortical interneurons appear to develop in parallel^18^. Although excitatory neurons are electrically coupled only during early development, interneurons maintain functional gap junctions into adulthood^10^,^15^,^18^,^21^. In fact, the coexistence of chemical and electrical synapses in interneurons of the mature cortex are important for promoting oscillatory rhythmic activity^10^,^11^,^13^,^15^,^22^ and generating synchronous activity^23^,^24^.

Layer 1 of the neocortex is ideal for studying interneuron circuit assembly as it has sparsely distributed interneurons^25^,^26^,^27^,^28^,^29^ that are connected by chemical as well as electrical synapses^22^,^30^,^31^. Recent evidence indicate that layer 1 interneurons not only play an important role in shaping the activity-dependent features of circuits in the deep layers of the neocortex^32^,^33^,^34^,^35^ but also integrate the feedback information from the thalamus^36^,^37^ and other cortical areas^38^. To date, while the synaptic organization of layer 1 interneurons has been extensively explored^30^,^31^, a systematic analysis of the functional development of circuits remains to be performed.

In this study, we characterize the developmental time course of GABAergic and electrical connections among layer 1 interneurons. We also quantitatively analyse the synaptic organization of layer 1 interneurons. Our data further reveal that the electrical coupling between layer 1 interneurons can promote their action potential (AP) generation and synchronous firing. We also demonstrate that electrically coupled layer 1 interneurons exhibit robust GABA-A receptor-mediated synchronous synaptic activity. Interestingly, disruption of Cx36-mediated electrical coupling between layer 1 interneurons severely impairs bidirectional GABAergic connections and affects excitatory synaptic inputs.

## Results

### Development of electrical and GABAergic connections

**Figure 1:**
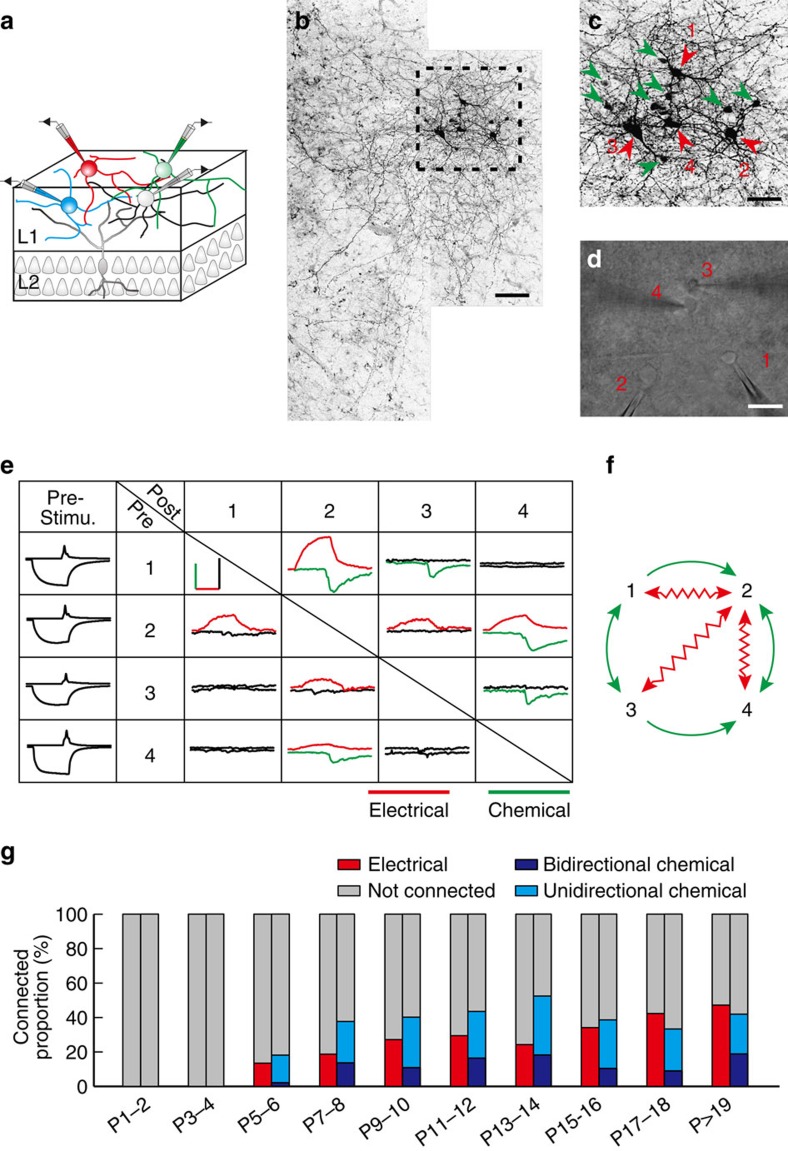
Development of electrical and GABAergic connections between interneurons in neocortical layer 1. (**a**) A schematic diagram of a quadruple whole-cell recording of four neurons in cortical layer 1. (**b**) An image of four layer 1 neurons filled with neurobiotin during recording and labelled with fluorescence-conjugated avidin. Scale bar, 100 μm. (**c**) A magnified image of **b** (dotted rectangular region). Red arrowheads indicate the four recorded neurons, and green arrowheads indicate dye-coupled neurons. Scale bar, 50 μm. (**d**) A DIC image of quadruple whole-cell recording of the four neurons in layer 1 shown in **b** and **c**. Scale bar, 50 μm. (**e**) Summary of the synaptic connections detected in this quadruple recording. The average traces of the postsynaptic responses are shown in the rectangle. Red traces indicate the existence of electrical synapses, and green traces indicate the existence of chemical synapses. Sample traces of AP and hyperpolarization potentials are shown to the left. Scale bar, 40 pA (green vertical scale bar), 20 ms (red horizontal scale bar), 200 mV (black vertical scale bar). ‘Pre-Stimu.', presynaptic potential; ‘Pre', presynaptic neuron; ‘Post', postsynaptic neuron. (**f**) A schematic diagram showing connections between the four neurons in **e**. Wavy red arrowheads indicate electrical connections, and green arrowheads indicate chemical connections. (**g**) Summary of proportion of electrical connections (red bars) and unidirectional/bidirectional chemical connections (light blue bars for unidirectional chemical connections and dark blue bars for bidirectional chemical connections) between interneuron pairs in neocortical layer 1 at different postnatal stages.

Layer 1 interneurons expand their neurites horizontally^26^,^27^,^38^. To preserve neurites, we prepared whole-mounts of the somatosensory cortex from GAD67-green fluorescent protein (eGFP) transgenic mice aged postnatal days 1–5 (P1-5) and acute horizontal slices of the somatosensory cortex from mice aged P6-25 (Supplementary Fig. 1). We identified neocortical layer 1 on the basis of the sparsely distributed cells. Guided by infrared differential interference contrast (DIC) and epifluorescence illumination, we simultaneously recorded from four layer 1 interneurons whose cell bodies were between 30 and 150 μm apart (the distance between the centres of cell bodies) (Fig. 1a,d). Compared with parasagittal sections of the brain^30^,^31^, arborizations of layer 1 interneurons, filled with neurobiotin during the recording, covered a larger field in the horizontal slice preparations (Fig. 1b), indicating that the horizontal slice preparation preserved anatomical and functional connectivity in neocortical layer 1. Furthermore, we found that layer 1 interneurons exhibited tracer coupling (neurobiotin, Fig. 1c), as observed in interneurons of other brain regions^39^,^40^. As in our previous study^26^, we found the vast majority of layer 1 neurons were positive for interneuron markers (Supplementary Fig. 2).

Once all four recordings were established, serial APs and hyperpolarization were sequentially triggered in one of the four neurons and the postsynaptic responses were then measured in the other three neurons to test chemical and electrical synapse formation between them. As shown in Fig. 1e and Supplementary Fig. 3, APs in the presynaptic neurons induced GABA-receptor-mediated inward currents in the postsynaptic neurons (green lines in Fig. 1e), while hyperpolarization induced electrical coupling-mediated outward currents (red lines in Fig. 1e). Although inward currents were induced by APs in both the chemically and electrically connected interneuron pairs, the GABA-receptor-mediated responses were distinguished based on their characteristic slow decay time course. To further confirm this, we examined the effects of bicuculline (BIC, 10 μM), a specific GABA-A receptor inhibitor. As expected, bicuculline treatment not only strongly reduced the inward current amplitudes but also completely eliminated the slow decay time responses (Supplementary Fig. 4a), suggesting that the latter are mediated by the GABA-A receptor. Furthermore, the treatment of carbenoxolone (CBX, 100 μM), a gap junction blocker, abolished hyperpolarization-induced outward currents (Supplementary Fig. 4b). Previous studies have shown that GABA-A and GABA-B receptors are both involved in mediating inhibitory synaptic response in neocortical layer 1 (refs 31, 35). However, single presynaptic APs barely induced GABA-B receptor-mediated postsynaptic response^31^,^35^, indicating that the inhibitory synaptic responses are mainly mediated by GABA-A receptors.

To further systematically study the development of electrical and GABAergic connections between layer 1 interneurons, we examined 947 pairs of layer 1 interneurons at different developmental stages (Fig. 1g and Supplementary Table 1). Our results showed that electrical and GABAergic connections (including unidirectional and bidirectional GABAergic connections) between layer 1 interneurons emerged at about the same developmental period (P5-6). The occurrence of electrical and GABAergic connections steadily increased during the second postnatal week, suggesting that the second postnatal week is a critical period for the development of synaptic connections between layer 1 interneurons. Together, these results suggest that the electrical and GABAergic connections between layer 1 interneurons have similar developmental time courses. Of note, we did not detect chemical or electrical connections between Cajal–Retzius cells, or between Cajal–Retzius cells and layer 1 interneurons at the early postnatal period.

### Microcircuitry between layer 1 interneurons

**Figure 2:**
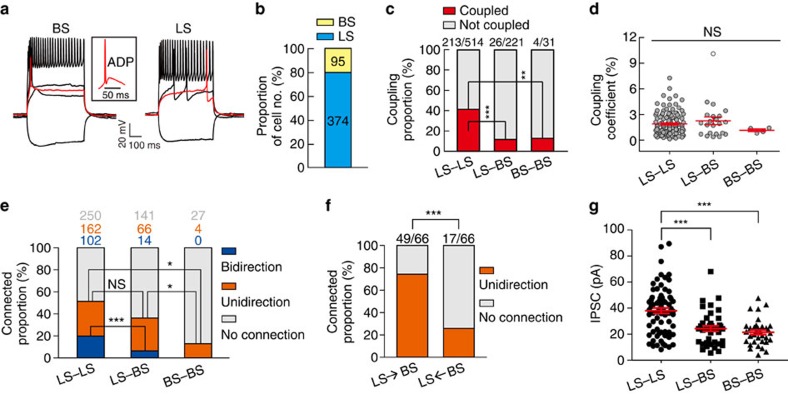
Electrical and GABAergic connections between layer 1 interneurons. (**a**) Representative traces of voltage responses to 500 ms current pulse step injections recorded in the current-clamp mode. The red traces show the initial AP spike. Layer 1 interneurons were divided into two subtypes, LS and BS neurons, based on their AP firing patterns. The inset shows the ADP in the initial AP of a BS neuron. (**b**) Histogram showing that the majority of interneurons displayed LS firing pattern (LS, 79.7%, 374 cells; BS, 20.3%, 95 cells; *n*=63 mice). (**c**) Summary of the proportion of electrical coupling observed between layer 1 interneurons at P9–P25. The rate of electrical connections between LS interneurons is significantly higher than the rate between LS and BS interneurons and between BS interneurons. (**d**) The scatter plot of coupling coefficients revealed no significant difference between interneuron subtypes (LS–LS pairs, 1.8±0.12%, *n*=122; LS–BS pairs, 2.2±0.45%, *n*=22; BS–BS pairs, 1.1±0.15%, *n*=4). (**e**) Summary of the proportion of GABAergic connections between interneuron subtypes. (**f**) The unidirectional chemical synapses between LS and BS interneurons showed directional selectivity. (**g**) The amplitude of uIPSCs between LS interneuron pairs (37.98±2.13 pA, *n*=73) was significantly larger than those between LS–BS pairs (24.22±2.09 pA, *n*=39) and BS–BS pairs (21.44±1.73 pA, *n*=34) (not including failures). *P<0.05, **P<0.01, ***P<0.001, *n.s*., *P*>0.05, not significant. χ^2^-test, Fisher's exact test and Mann–Whitney rank sum test. Error bars in **d** and **g** represent mean±s.e.m. ADP, after depolarization.

On the basis of their electrophysiological properties, layer 1 interneurons were classified into two subtypes (Fig. 2a), burst spiking (BS) and late spiking (LS), as shown previously^26^,^41^,^42^. The key differences between them were a delay in the initial spike, spike firing pattern and after depolarization (the inset of Fig. 2a). LS neurons were identified by a delay with a steady ramp depolarization leading up to the initial spike at threshold current injections. BS neurons fired a burst of APs at the initial spike without any delay, and after depolarizations were only observed in BS neurons. Out of a total of 469 layer 1 interneurons obtained from P9-25 mice, the number of LS and BS interneurons was 374 (79.7%) and 95 (20.3%), respectively (Fig. 2b).

We further analysed electrical coupling between layer 1 interneurons in the developing neocortex at P9-25. Of the LS pairs, we found 41.4% were electrically coupled (Fig. 2c). By contrast, only 11.8% of LS–BS pairs and 12.9% of BS pairs were coupled (Fig. 2c). These results indicate that electrical connections exhibit cell-type selectivity among neocortical layer 1 interneurons, as shown previously^30^. A comparison of the coupling coefficients between coupled pairs revealed no significant difference between interneuron subtypes (Fig. 2d). Moreover, the estimated bidirectional coupling coefficients of each coupled pair upon current injection to cell 1 or cell 2 showed no significant differences (Supplementary Fig. 5a). Furthermore, although the coupling coefficient did not depend on the distance between the somata of layer 1 interneurons (*P*>0.05, R^2^=0.0182, Supplementary Fig. 5b), the proportion of electrical connectivity is significantly reduced with increased distance between interneuron somata (Supplementary Fig. 5c).

Both unidirectional and bidirectional GABAergic connections were abundant between layer 1 interneurons at P9–P25 (Fig. 2e). The three-dimensional morphological reconstruction showed that the mean number of potential synaptic contacts established by LS–LS interneurons was 11.2±2.1 (*n*=5) (Supplementary Fig. 6). Unidirectional GABAergic connections were significantly more numerous in LS–LS pairs and LS–BS pairs compared with BS–BS pairs (LS–LS pairs, 31.5% (162 out of 514); LS–BS pairs, 29.9% (66 out of 221); BS–BS pairs, 12.9% (4 of 31); and *P*<0.05, Fig. 2e). Remarkably, the incidence of bidirectional chemical synapses between LS–LS pairs (19.8%, 102 out of 514) was significantly higher than between LS–BS pairs (6.3%, 14 out of 221, *P*<0.001) and BS–BS pairs (0%, 0 out of 31, *P*<0.01) (Fig. 2e). In addition, the unidirectional chemical synapses between LS and BS interneurons showed directional selectivity. Around 74.2% of unidirectional pairs formed connections from LS to BS, whereas only 25.8% formed connections from BS to LS (*P*<0.001, Fig. 2f). Moreover, the peak amplitude of unitary inhibitory postsynaptic current (uIPSCs) between LS interneuron pairs was significantly larger than that between LS–BS pairs and BS–BS pairs (Fig. 2g). Overall, our results demonstrate that distinct subtypes of layer 1 interneurons exhibited highly specific synaptic connections.

**Figure 3:**
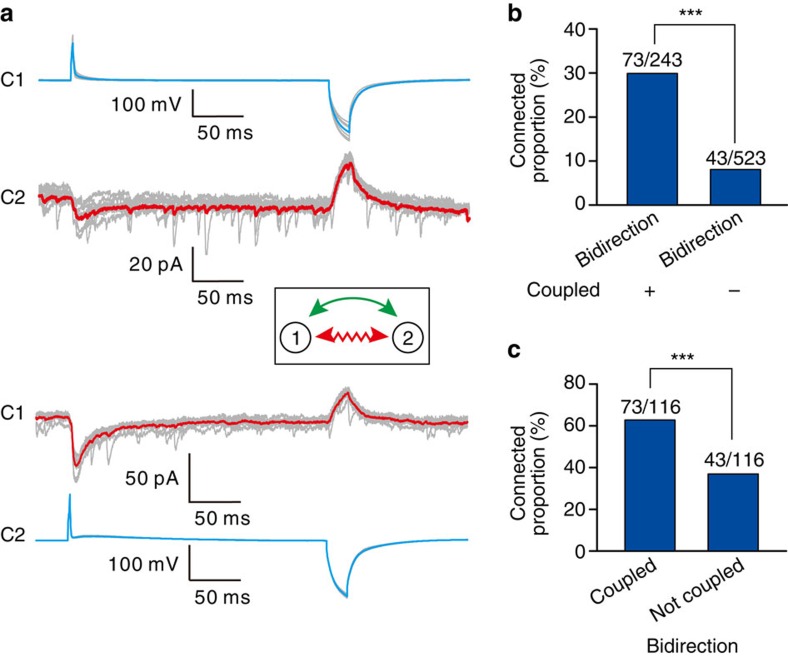
Preferential existence of bidirectional chemical connections in electrically coupled pairs. (**a**) A representative sample pair with bidirectional chemical connection and reciprocal electrical connection between two layer 1 interneurons. APs and hyperpolarization sequentially triggered in the presynaptic neurons (blue traces) and responses (red traces) of GABAergic connections (inward currents) and electrical connections (outward currents) recorded in the postsynaptic neurons. The bold traces represent the average and the grey traces represent the individual traces. A schematic diagram showing electrical and GABAergic connections between the two neurons (inset). Wavy red arrowheads indicate electrical connections, and green arrowheads indicate GABAergic connections. (**b**) Bidirectional chemical connections between layer 1 interneurons preferentially existed in electrically coupled pairs. (**c**) Layer 1 interneuron pairs connected by bidirectional chemical synapses preferentially formed electrical synapses. ***P<0.001, χ^2^-test.

Interestingly, bidirectional chemical connections between layer 1 interneurons existed preferentially in electrically coupled pairs (Fig. 3). Around 30% of electrically coupled pairs, versus only 8.2% of uncoupled pairs, had bidirectional GABAergic connections (*P*<0.001, Fig. 3b). Moreover, in interneuron pairs bidirectionally connected by GABAergic synapses, the percentage of electrical coupling (62.9%) was significantly higher than in the non-coupled pairs (37.1%,) (*P*<0.001, Fig. 3c). These results suggest that electrical coupling-mediated communication correlates with the formation of bidirectional GABAergic connections in neocortical layer 1 interneurons.

### Bidirectional chemical synapses affect firing synchrony

**Figure 4:**
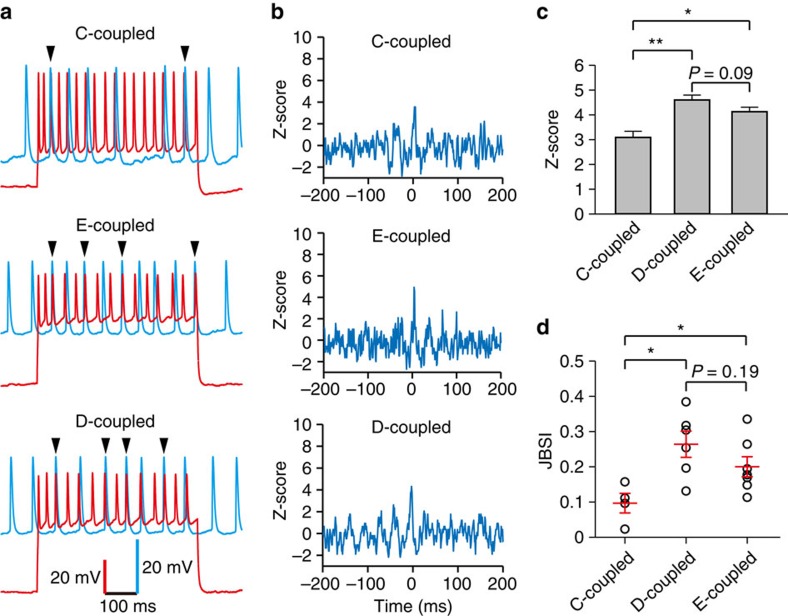
The effect of bidirectional chemical synapse in controlling firing synchrony between layer 1 interneurons. To test for firing synchrony in three interneuron groups (C-coupled, E-coupled and D-coupled), paired spike trains were elicited by suprathreshold current in the driver cell (red traces, 30–50 Hz) and by tonic suprathreshold depolarization in the follower cell (blue traces, ∼10 Hz), respectively. (**a**) Sample traces of paired spike trains from two neurons coupled by different patterns of connectivity in neocortical layer 1, the arrowheads indicate the synchronous spikes. (**b**) Normalized cross-correlogram (*Z*-score) for neuron pairs in (**a**). Bin size is 2 ms. Note that the cross-correlogram exhibited a peak ∼0 ms in all three groups. (**c**) Average *Z*-scores for different patterns of connectivity. Note that *Z*-scores of E- and D-coupled pairs were significantly higher than that of C-coupled pairs, and *Z*-scores of D-coupled pairs were slightly lower than that of E-coupled pairs. (**d**) Average JBSI values for different patterns of connectivity. C-coupled pairs=4, E-coupled pairs=6, D-coupled pairs=7, from 10 mice. *P<0.05, **P<0.01, two-tailed paired *t*-test. Error bars represent mean±s.e.m.

The bidirectional chemical coupling between interneurons was shown to produce synchronous or anti-synchronous activity^43^,^44^. We thus tested firing synchrony between layer 1 interneurons at P15-20 (Fig. 4). Paired interneurons communicating only through bidirectional chemical synapses are referred to as chemically coupled pairs (C-coupled), pairs communicating only through electrical synapses as electrically coupled pairs (E-coupled), and pairs communicating through both bidirectional chemical and electrical synapses as dually coupled pairs (D-coupled). To examine firing synchrony and compare across the three groups (C-, E- and D-coupled pairs), we evoked firing in interneuron pairs by injecting periodic currents steps (600 ms steps at 5 s intervals) into one cell to trigger firing at γ-frequency (Fig. 4a, driver cell, red traces, ∼30–50 Hz) and tonic suprathreshold current into the other cell (Fig. 4a, follower cell, blue traces, ∼10 Hz). Synchronous spikes could be observed in all three groups (Fig. 4a, arrowheads at the top of the traces).

To quantify the observed synchrony, we computed the normalized spike cross-correlogram (Z-score), and found that it exhibited a peak ∼0 ms in all three groups (Fig. 4b, bin size=2 ms). In C-coupled pairs, the average *Z*-score was 3.09±0.25 (*n*=4). In comparison, the average *Z*-scores of E-coupled and D-coupled pairs was significantly higher (4.61±0.24 for E-coupled pairs, *P*<0.01, *n*=6; 4.13±0.18 for D-coupled pairs, *P*<0.05, *n*=7) (Fig. 4c). To further validate spiking synchrony, we also used the Jitter-Based Synchrony Index (JBSI) to quantify the strength of synchrony^45^. Similar to the *Z*-score analysis, the average JBSI value of E-coupled and D-coupled pairs was significantly higher than that of C-coupled pairs (0.10±0.03 for C-coupled pairs; 0.26±0.04 for E-coupled; and 0.20±0.03 for D-coupled pairs; *P*<0.05, Fig. 4d). Of note, we found that both mean *Z*-score and JBSI of D-coupled pairs appeared to be slightly lower than those of E-coupled pairs (Fig. 4c,d). These data indicated that synchrony driven by dual coupling was relatively lower than that driven by electrical coupling alone at 40 Hz γ-frequency.

### Synchronous synaptic activity between layer 1 interneurons

**Figure 5:**
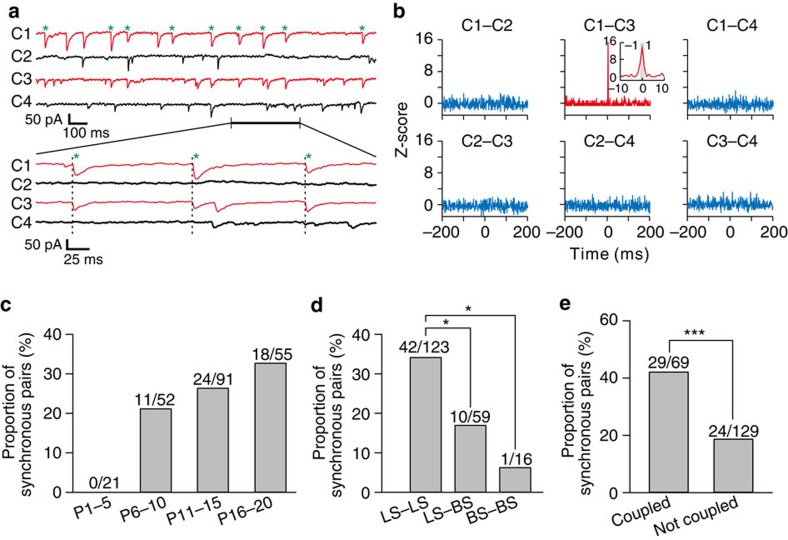
Synchronous synaptic activity between layer 1 interneurons. (**a**) Sample traces of spontaneous activity of four layer 1 interneurons. High-temporal-resolution displays of a segment of recordings (thick black line) are shown at the bottom. Green asterisks and dotted lines indicate synchronized events between two neurons (cell 1 and 3, red traces). (**b**) Normalized cross-correlogram (*Z*-score) for neuron pairs in **a**. Bin size is 1 ms. Note that the frequency of events is significantly increased at ∼0 ms (inset) for the interneuron pair (1 versus 3, red), but not for any other neuron pairs. The grey region in the inset corresponds to −1 ms≤Δ*t*≤1 ms. Similar symbols and displays are used in subsequent figures. (**c**) Proportion of layer 1 interneuron pairs exhibiting synchronized spontaneous activity at different developmental stages. Note that synchronous synaptic activity appeared to be correlated with the developmental process. (**d**) Proportion of layer 1 interneuron pairs of different subtypes from P6 to P20 exhibiting synchronized spontaneous activity. (**e**) Proportion of electrically coupled or not coupled interneuron pairs from P6 to P20 exhibiting synchronized spontaneous activity. *n*=47 mice. *P<0.05, ***P<0.001, χ^2^-test and Fisher's exact test.

Synchronous activity is an emergent property between interneurons^46^. However, the synchronization between interneurons is mainly observed in spiking activity^10^,^12^,^15^,^23^,^47^. Interestingly, synchronous spontaneous currents were frequently observed between layer 1 interneurons (Fig. 5a). To quantitatively assess this, we computed the cross-correlogram of spontaneous currents and found that synchronization of spontaneous currents between layer 1 interneuron pairs was ∼0 ms (within −1 to 1 ms) (Fig. 5b, inset). To test whether the observed synchronous activity is indeed synaptic in nature, we performed pharmacological experiments using bicuculline (10 μM) as well as D-APV (50 μM) and NBQX (10 μM), the inhibitors of *N*-methyl-D-aspartate-type and a-amino-3-hydroxy-5-methyl-4-isoxazolepropionic acid-type glutamate receptors, respectively. We found that bicuculline treatment completely eliminated spontaneous activity between layer 1 interneurons (Supplementary Fig. 7a–d). In contrast, D-APV and NBQX showed no significant effect (Supplementary Fig. 7e–h). Overall, these results demonstrate that layer 1 interneurons exhibit synchronous GABA-A receptor-mediated inhibitory synaptic activity.

Notably, synchronous synaptic activity appeared to be correlated with the developmental process (Fig. 5c). Moreover, we found that the rate of synchronous synaptic activity between LS interneurons was 34.1%, which is significantly higher than the rate between LS and BS interneurons (16.9%, *P*<0.05) and between BS interneurons (6.3%, *P*<0.05) (Fig. 5d). Interestingly, the percentage of synchronization between electrically coupled pairs (42.0%) was significantly higher than between non-coupled pairs (18.6%), suggesting that electrically coupled interneurons preferentially generate synchronous synaptic activity in neocortical layer 1 (Fig. 5e, *P*<0.001).

### Connexin 36 is required for the electrical coupling

**Figure 6:**
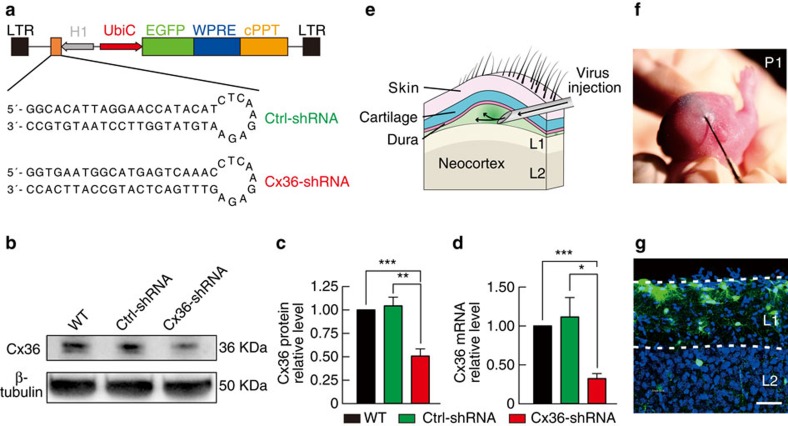
Inducible lentiviral shRNA mediates effective knockdown of Connexin 36. (**a**) Schematic illustration of the shRNA-expressing lentiviral constructs. The Ctrl-shRNA and the Cx36-shRNA inserts contained 21-nt sense and antisense strands. Sense and antisense strands were linked through a standard 5′- CTCAAGAGA -3′ loop structure specific to mammalian cells. (**b**) Western blot of protein samples isolated from cultured E18 mouse primary cortical neurons infected with Ctrl-shRNA and Cx36-shRNA lentiviruses or WT cells (uninfected cells) and probed with anti-Cx36 and anti-β-tubulin antiserum. β-Tubulin served as a loading control (*n*=4 pregnant mice). (**c**,**d**) Quantitative analysis of western blot (**c**) and real-time PCR (**d**) showed that both the protein (∼50%) and mRNA levels (∼68%) of Cx36 were significantly reduced in Cx36-shRNA-transfected cells as compared with WT cells and Ctrl-shRNA-transfected cells; no significant difference was noted between WT and Ctrl-shRNA groups (*P*>0.05). Cx36 mRNA expression levels normalized for *GAPDH*. (**e**) Schema showing the method of virus injection, using a tiny self-made glass injector to inject the virus into the gap between the dura and cortical layer 1 at P1 with CD-1 mice (also see Methods). (**f**) Image of virus injection into neocortical layer 1 at P1. The fast green injected along with virus helped in estimating the extent of diffusion of the virus in neocortical layer 1. (**g**) Representative distribution of lentivirus-labelled GFP-positive (GFP^+^) cells. GFP^+^ cells densely packed in neocortical layer 1, but sparsely distributed in deep layers, were detected at P15. Scale bar, 50 μm. *P<0.05, **P<0.01, ***P<0.001, two-tailed paired *t*-test. Error bars represent mean±s.e.m.

Cx36 is a major connexin isoform that mediates electrical coupling between interneurons in the neocortex^23^,^48^. Consistent with this, we found Cx36-positive puncta at the dendrodendritic contacts of two layer 1 interneurons which were labelled by *in vivo* injection of eGFP-expressing lentivirus at P1 (Supplementary Fig. 8). To investigate the roles of gap junctions in circuit development in neocortical layer 1, we engineered acute loss-of-function of Cx36 using RNA interference (RNAi). We designed lentiviral constructs expressing either control Cx36 sequence (scrambled sequence, Ctrl-shRNA) or Cx36-knockdown sequence (Cx36-shRNA), together with eGFP (Fig. 6a and Supplementary Fig. 9). To test the efficiency of RNAi, the Ctrl-shRNA and Cx36-shRNA lentiviruses were examined by western blot and quantitative real-time PCR in cultured mouse primary neurons (see Methods). Western blot and quantitative real-time PCR analysis showed that both the protein (∼50%) and mRNA levels (∼68%) of Cx36 were significantly reduced in Cx36-shRNA-transfected cells as compared with wild-type cells (WT, untransfected cells, *P*<0.001) and Ctrl-shRNA-transfected cells (*P*<0.05, Fig. 6b–d); no significant difference was observed between WT and Ctrl-shRNA groups (*P*>0.05, Fig. 6c,d). These results indicate that Cx36-shRNA effectively suppresses the expression of Cx36 in neurons.

**Figure 7:**
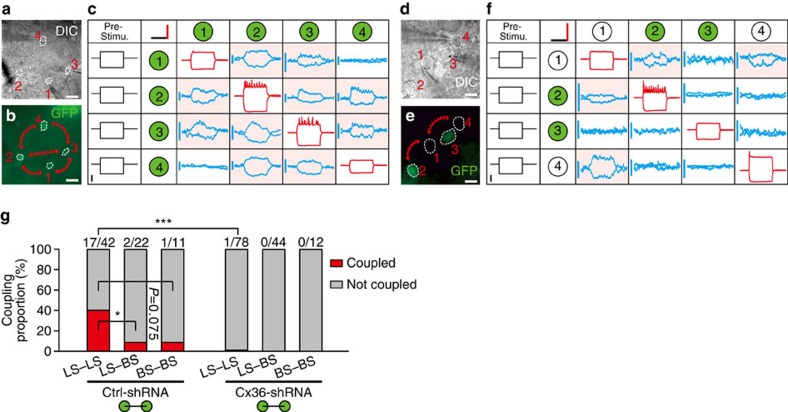
Connexin 36 is required for formation of electrical coupling between interneurons in neocortical layer 1. (**a**,**b**,**d**,**e**) DIC (**a**,**d**) and GFP expression (**b**,**e**) images of a quadruple recording of four layer 1 interneurons in a Ctrl-shRNA (**a**,**b**) and Cx36-shRNA (**d**,**e**) neocortical slice. The red arrows indicate reciprocal electrical coupling (**b**,**e**). Scale bar, 20 μm (**a**,**b**), 40 μm (**d**,**e**). (**c**,**f**) Sample traces of voltage changes in the four neurons in response to sequential current injection into one of the four neurons in Ctrl-shRNA slices (**c**) and Cx36-shRNA slices (**f**). Green circles indicate GFP^+^ cells, and white circles indicate GFP-negative (GFP^−^) cells. The average traces are shown in individual table cells at varying scales. Scale bar, 3 mV (blue), 70 mV (red), 200 ms (horizontal, black) and 200 pA (vertical, black). ‘Pre-Stimu.', presynaptic stimulus protocol. (**g**) Summary of the proportion of electrical coupling observed between layer 1 GFP^+^ interneurons in Ctrl-shRNA or Cx36-shRNA slices at P9–P25. Green circles indicate GFP^+^ cells. *n*=21 mice for Ctrl-shRNA group, 37 mice for Cx36-shRNA group. ***P<0.001, χ^2^-test, Fisher's exact test.

We next examined the functional effects of Cx36 downregulation on electrical coupling between layer 1 interneurons. Ctrl-shRNA and Cx36-shRNA lentiviruses were horizontally injected into the gap between dura and necortical layer 1 in WT neonatal mice (P1), until the viral solution was extensively diffused (Fig. 6e,f, see Methods). As shown in Fig. 6g, dense GFP-positive (GFP^+^) cells in neocortical layer 1, but sparse GFP^+^ cells in deep layers, were detected at P15, indicating that intracranial injection of virus can effectively infect neurons in neocortical layer 1. To test electrical coupling between layer 1 interneurons, we performed quadruple whole-cell recordings on Ctrl-shRNA slices (Fig. 7a–c) and Cx36-shRNA slices (Fig. 7d–f) in the developing neocortex at P9–P25. Because the proportion of electrical coupling was dependent on the distance between the recorded somata of interneurons (Supplementary Fig. 5c), we kept the distances between GFP^+^ neurons as consistent as possible in Ctrl-shRNA and Cx36 knockdown groups (Supplementary Fig. 10). Once all four recordings were established, hyperpolarizing and depolarizing step currents were injected sequentially into one of the four neurons, and subsequent voltage changes were monitored in all four neurons to probe gap junction-mediated electrical coupling (Fig. 7c,f). We found that expression of Ctrl-shRNA had no discernible effect on electrical coupling, but expression of Cx36-shRNA almost eliminated the electrical coupling between layer 1 interneurons (0.7%, 1 out of 134 pairs, Fig. 7g). Our results clearly demonstrate that Cx36 is required for the formation of functional electrical coupling between layer 1 interneurons.

### Electrical coupling affect bidirectional chemical synapses

**Figure 8:**
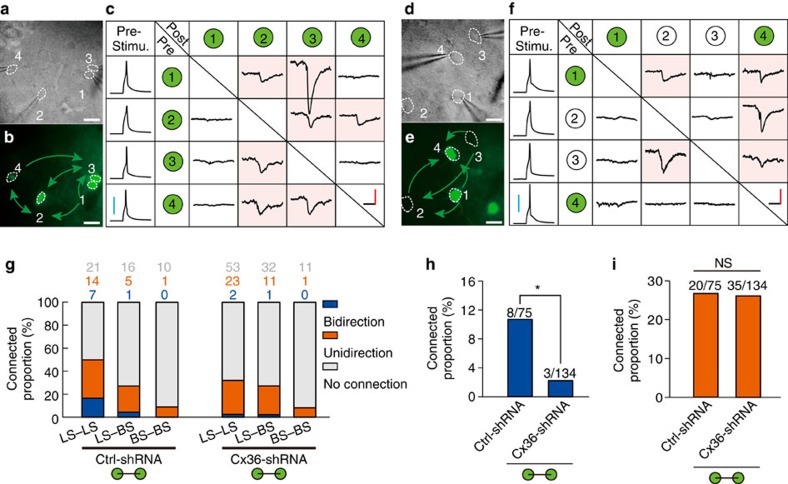
Electrical coupling is required for the formation of bidirectional chemical synapses between layer 1 interneurons. (**a**–**f**) Quadruple whole-cell recordings of four layer 1 interneurons in a Ctrl-shRNA (**a**–**c**) and Cx36-shRNA (**d**–**f**) slice. DIC (**a**,**d**) and fluorescence images (**b**,**e**) of the respective quadruple whole-cell recordings are shown. The green arrows in **b** and **e** indicate the direction of chemical synapses. A summary of the chemical synaptic connections detected in the quadruple recordings is also shown (**c**,**f**). Pink shading indicates the existence of chemical synapses. Scale bar, 20 μm in (**a**,**b**,**d**,**e**), 20 pA (red), 40 mV (blue) and 15 ms (black) in (**c**,**f**). ‘Pre-Stimu.', presynaptic potential; ‘Pre', presynaptic neuron; ‘Post', postsynaptic neuron. (**g**) Summary of the proportion of chemical synapse formation between layer 1 interneurons in Ctrl-shRNA (GFP^+^-GFP^+^ pairs) and Cx36-shRNA (GFP^+^-GFP^+^ pairs) slices at P9–P25. Green circle indicates GFP^+^ cells. (**h**) The proportion of bidirectional chemical connections in Cx36-shRNA group was significantly less than in Ctrl-shRNA groups. (**i**) The proportion of unidirectional chemical connections in Ctrl-shRNA and Cx36-shRNA groups showed no significant differences. *n*=21 mice for Ctrl-shRNA group, 37 mice for Cx36-shRNA group. *P<0.05, n.s., not significant, *P*>0.05. χ^2^-test, Fisher's exact test.

Having found that synaptogenesis of electrical and GABAergic connections of interneurons in neocortical layer 1 exhibit a similar developmental time course, we then tested whether electrical coupling modulates GABAergic connections of layer 1 interneurons. We performed quadruple whole-cell recordings on horizontal slices expressing Ctrl-shRNA (Fig. 8a–c) or Cx36-shRNA (Fig. 8d–f) to test GABAergic connections between layer 1 interneurons in the developing neocortex at P9–P23. We found that GABAergic connections were not significantly different between Ctrl-shRNA and Cx36-shRNA groups (37.3% (28 out of 75) in Ctrl-shRNA; 28.4% (38 out of 134) in Cx36-shRNA; *P*=0.21, Fig. 8g). However, the percentage of bidirectional chemical connections in the Cx36-shRNA group (2.2%) was significantly less than in the Ctrl-shRNA group (10.7%, *P*<0.05) (Fig. 8h). Given that bidirectional chemical connections between layer 1 interneurons existed preferentially in electrically coupled pairs (Fig. 3), these results strongly indicate that electrical transmission is required for the development of bidirectional GABAergic chemical synapses between these neurons. In contrast, no significant differences were observed in the unidirectional chemical connections between Ctrl-shRNA (26.7%) and Cx36-shRNA (26.1%) groups (Fig. 8i). In addition, there were no significant differences in the amplitude of uIPSCs between the Ctrl-shRNA and Cx36-shRNA groups (Supplementary Fig. 11). Notably, the interneurons expressing Cx36-shRNA exhibited normal morphological and intrinsic electrophysiological properties (Supplementary Fig. 12).

### Modulation of miniature postsynaptic currents

**Figure 9:**
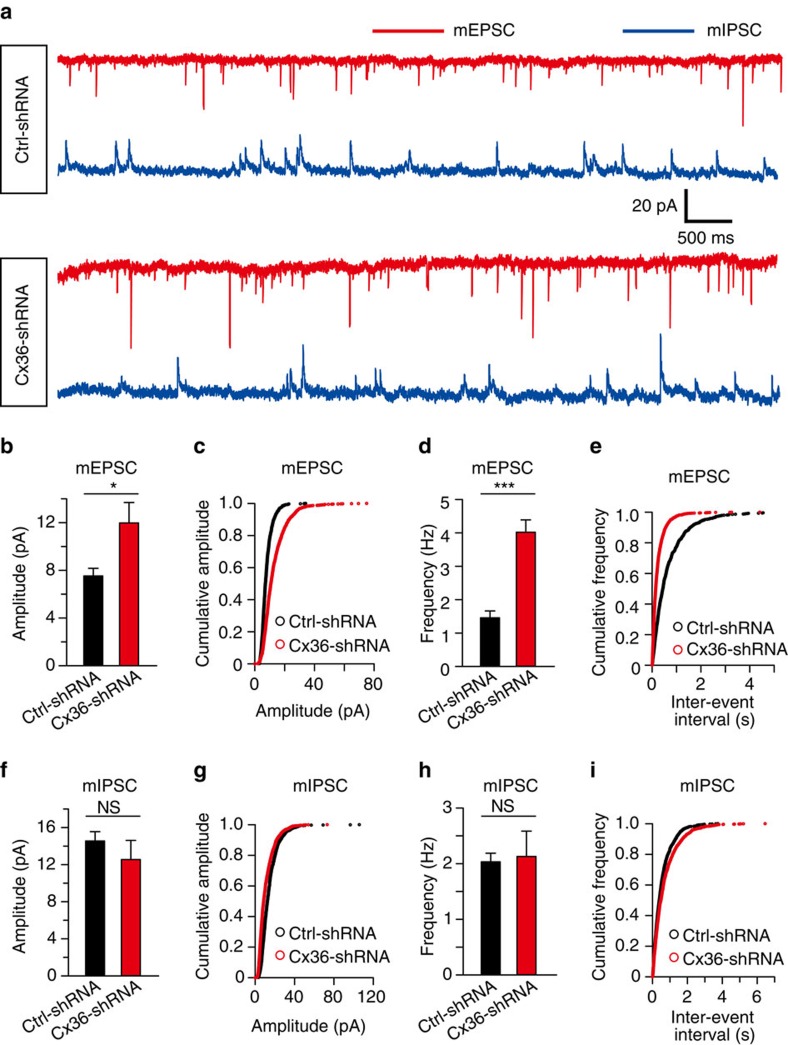
Modulation of miniature postsynaptic currents by electrical coupling in neocortical layer 1. (**a**) Representative traces of inward mEPSCs (red traces, holding potential of −60 mV) and outward mIPSCs (blue traces, holding potential of +10 mV) recorded in Ctrl-shRNA and Cx36-shRNA groups in the presence of tetrodotoxin (5 μM). (**b**–**e**) Histograms (**b**,**d**) and cumulative distributions (**c**,**e**) of mEPSC amplitudes (**b**,**c**) and frequencies (**d**,**e**). Both the peak amplitude and frequency of mEPSCs in Cx36-shRNA group were significantly higher than those in Ctrl-shRNA group. (**f**–**i**) Histograms (**f**,**h**) and cumulative distributions (**g**,**i**) of mIPSC amplitudes (**f**,**g**) and frequencies (**h**,**i**). The peak amplitude and frequency of mIPSCs showed no significant differences between Cx36-shRNA and Ctrl-shRNA groups (*n*=12 in Cx36-shRNA group, *n*=8 in Ctrl-shRNA group, three mice for each group). *P<0.05, ***P<0.001, n.s., *P*>0.05, not significant. Two-tailed paired *t*-test. Error bars represent mean±s.e.m.

To test whether synaptic transmission in neocortical layer 1 is altered by knockdown of Cx36, we examined the amplitude and frequency of miniature EPSCs (excitatory postsynaptic current) (mEPSCs) and miniature IPSCs (mIPSCs) in interneurons from P15 to P20. Inward mEPSCs (holding potential of −60 mV) and outward mIPSCs (holding potential of +10 mV) were recorded sequentially in the same neuron in the presence of tetrodotoxin (TTX, 5 μM). Representative traces of mEPSCs (red trace) and mIPSCs (blue trace) recorded in Ctrl-shRNA and Cx36-shRNA groups were shown in Fig. 9a. We found that both the peak amplitude and frequency of mEPSCs in the Cx36-shRNA group were significantly higher than in the Ctrl-shRNA group (Fig. 9b–e and Supplementary Table 2). In contrast, mIPSCs showed no significant differences between Cx36-shRNA group and Ctrl-shRNA group (Fig. 9f–i and Supplementary Table 2). These results suggest that there is a facilitation of excitatory synaptic transmission onto interneurons in neocortical layer 1 after the elimination of electrical coupling.

### Electrical coupling promotes synchronous firing

**Figure 10:**
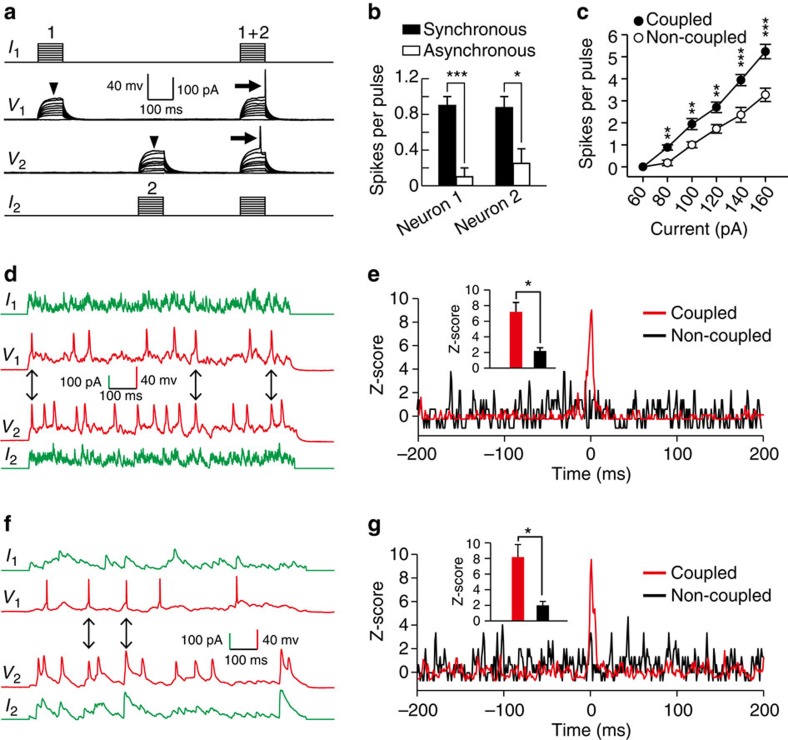
Electrical coupling promotes AP generation and synchronous firing of layer 1 interneurons. (**a**) Sample traces of synchronous (1+2) or asynchronous (1 or 2) injection of subthreshold current pulses into electrically coupled interneurons in neocortical layer 1. Note that synchronous injection, but not asynchronous injection (arrowheads), results in AP generation (arrows). (**b**) Summary of the firing rate of the two interneurons shown in **a** responding to 80 pA current injection. (**c**) Summary of the firing rate in electrically coupled or non-coupled interneurons responding to simultaneous current injections. (**d**–**g**) Electrical coupling promoting synchronous firing of electrically coupled neurons in neocortical layer 1 in response to uncorrelated simulated neuronal activity (**d**,**e**) or uncorrelated native neuronal activity (**f**,**g**). (**d**,**f**) Sample traces of voltage changes in electrically coupled interneurons. Arrows indicate the spikes that occur in both neurons within a 1 ms window. (**e**,**g**) Normalized cross-correlogram (*Z*-score) analysis. The bin size is 1 ms. Note that the firing frequency is significantly increased near 0 ms for coupled interneuron pairs (red) but not for non-coupled interneuron pairs (black), indicating synchronous firing. The insets show the statistical analysis of *Z*-scores of coupled/non-coupled interneuron pairs. *P<0.05, **P<0.01, ***P<0.001. Mann–Whitney rank sum test and two-tailed paired *t*-test. Error bars represent mean±s.e.m.

It has long been postulated that synchronous neuronal activity facilitates chemical synapse formation and neuronal circuit assembly^49^,^50^. Therefore, we hypothesize that electrical coupling in early postnatal stage facilitates synchronized activity, which in turn promotes bidirectional chemical synapse formation, between layer 1 interneurons. To test this, we first investigated whether electrical coupling facilitates neuronal activity of layer 1 interneurons at P6-9. Subthreshold depolarizing current pulses were injected at different times or at the same time into two interneurons in neocortical layer 1. In electrically coupled pairs, the asynchronous subthreshold pulses did not generate an AP in either neuron (Fig. 10a, arrowheads, and Fig. 10b, open bars; neuron 1, 0.10±0.10 spikes per pulse; neuron 2, 0.25±0.16 spikes per pulse). However, when the same current pulses were synchronously injected into both neurons, the two neurons reached AP threshold and generated spikes (Fig. 10a, arrows, and Fig. 10b, filled bars; neuron 1, 0.90±0.10 spikes per pulse; neuron 2, 0.88±0.13 spikes per pulse). These observations were consistent across additional pairs of electrically coupled versus non-coupled interneuron pairs (Fig. 10c; coupled pairs, *n*=17; non-coupled pairs, *n*=11; *P*<0.01). The results indicate that electrical coupling can strongly promote the generation of APs in coupled interneurons in neocortical layer 1.

We next determined whether electrical coupling could facilitate synchronous spiking of interneurons in response to natural stimuli. First, we simulated natural activity by injecting two electrically coupled or non-coupled layer 1 interneurons with uncorrelated random current signals obtained by convolving the Poisson trains of recorded spontaneous excitatory postsynaptic current waveforms (Fig. 10d; *I*_1_ and *I*_2_)^10^. Second, we directly recorded the spontaneous subthreshold activity of layer 1 interneurons at P5-8 and then injected two electrically coupled or non-coupled interneurons with signals corresponding to the recorded uncorrelated native neuronal activity (*I*_1_ and *I*_2_; Fig. 10f). We then analysed the cross-correlogram of firing induced in these layer 1 interneurons and found that there was a significant increase in firing centred ∼0 ms in coupled pairs compared with non-coupled pairs (Fig. 10e, coupled, *n*=6; non-coupled, *n*=4; *P*<0.05; and Fig. 10g, coupled, *n*=5; non-coupled, *n*=4; *P*<0.05). These results suggest that gap junction-mediated electrical coupling between interneurons in neocortical layer 1 can effectively promote precise (within 1 ms) synchronous firing in response to uncorrelated neuronal activity. Notably, disruption of electrical coupling by Cx36 shRNA expression strongly impaired the synchronous firing between layer 1 interneurons (Supplementary Fig. 13). Given that bidirectional chemical coupled interneurons preferentially form gap junction-mediated electrical coupling (Fig. 3), which promotes AP generation and synchronous firing, this process could be a mechanism by which bidirectional chemical synapses form preferentially between electrically coupled interneurons.

## Discussion

In this study, we observed that electrical and chemical synapses between neocortical layer 1 interneurons had a parallel developmental time course and demonstrated that distinct subtypes of layer 1 interneurons developed highly specific electrical and GABAergic connections. Furthermore, we found that electrical connections effectively promote AP generation and synchronous firing in neocortical layer 1. Moreover, we observed that layer 1 interneurons exhibit robust synchronous GABA-A-receptor-mediated synaptic activity, which preferentially develops between electrically coupled pairs. Notably, our results revealed that electrical transmission between layer 1 interneurons was required for the development of precise bidirectional inhibitory synapses between these neurons. To our knowledge, our results are the first to provide the direct evidence that electrical coupling modulates microcircuit formation in neocortical interneurons.

It is uncertain precisely when interneurons form GABAergic and electrical connections during dvelopment. For example, studies using paired recordings showed that electrical coupling is present among inhibitory neurons in the thalamic reticular nucleus in newborn mice, whereas inhibitory chemical synapses were detected after postnatal day 4 (ref. 51). In contrast, we observed that electrical and GABAergic connections between layer 1 interneurons appear at about the same developmental period (P5-6), and these connections steadily increase during the second postnatal week. Consistent with a recent report^18^, these results indicate that electrical and chemical synapses between neocortical interneurons have a parallel developmental time course. It will be interesting to further investigate the time course of electrical synapse development of other interneurons in the neocortex.

Furthermore, we found that 41.4% of LS–LS pairs were electrically coupled during P9-23, whereas only 11.8% of LS–BS pairs and 12.9% of BS–BS pairs were electrically coupled. These data indicate that electrical synapses are preferentially formed between LS interneurons. We also noted that the proportion of electrical coupling in LS–LS pairs (41.4%) in this study is lower than previously reported (83%) (ref. 30). Given that the rate of electrical connections is significantly reduced with increased distance between interneuron somata (Supplementary Fig. 5c), we reason that the distance between recorded neurons in this study (quadruple recordings) is farther than in previous studies (pair recordings)^30^.

Previous reports indicated that the morphological characteristics of LS neurons in layer 1 are similar to those of neurogliaform cells, and both dendrites and axons of LS neurons are included primarily in layer 1 (refs 26, 30, 42, 52). In contrast, BS neurons normally exhibit bipolar or bitufted morphology and have long axonal branches descending downward to the deeper layers^26^,^42^,^53^. Building on these reports and our results (Fig. 2), we attempted to construct a model to explain the architectural features of microcircuits in layer 1. LS neurons preferentially form local inhibitory connections between themselves and modulate the activity of BS neurons by providing inhibition to them or by direct effect on the apical dendrites of projection neurons located in layer 1. In contrast, BS neurons usually transform the inhibition to neurons in deep layers through their descending axons. This mode of microcircuit assembly in layer 1 has been noted in the rat neocortex^33^.

Interestingly, we found that interneurons in layer 1 exhibit robust synchronous spontaneous and evoked synaptic activity mediated by GABA-A receptors. Although synchrony is most commonly attributed to electrical coupling, accumulated evidence suggests that it is based on both electrical and chemical synapses^10^,^15^,^23^,^54^. For example, synchrony of spiking in the absence of electrical coupling was nearly unchanged after blocking ionotropic glutamate receptors, but was profoundly reduced after blocking GABA-A receptors^55^, suggesting that inhibitory postsynaptic potentials were both necessary and sufficient for synchrony between cortical interneurons. Indeed, we found that layer 1 interneurons exhibit GABA-A receptor-mediated synchronous synaptic activity.

In this study, we took advantage of RNAi technology to reduce the expression of Cx36 in neocortical layer 1 postnatally. Our data show that Cx36-shRNA significantly reduced protein and mRNA expression of Cx36 in cultured neurons (Fig. 6c,d). The incomplete knockdown efficiency might be partly due to the efficacy of lentiviral transduction of cultured neurons. Given the expression of Cx36-shRNA almost completely eliminated electrical coupling between layer 1 interneurons (0.7%, 1 out of 134 pairs), it demonstrates that the Cx36 knockdown approach is specific and effective. In addition, we developed a novel method to inject control or Cx36 knockdown lentiviruses specifically into neocortical layer 1 (see Methods, Fig. 6e–g). Similar to the Cx36 knockout mice^23^,^54^,^56^, we found that Cx36 knockdown produces a drastic loss of electrical coupling between interneurons, indicating that Cx36 is critical for the formation of functional electrical synapses between interneurons in neocortical layer 1. Unexpectedly, we observed that Cx36 knockdown can dramatically reduce the bidirectional, but not the unidirectional, inhibitory connections, between layer 1 interneurons. Moreover, we found that bidirectional inhibitory connections preferentially develop between electrically coupled interneuron pairs. These results demonstrate that gap junctions between layer 1 interneurons are required for the formation of bidirectional inhibitory connections. Of note, we found that the fraction of bidirectional chemical connection in the absence of electrical coupling in the Cx36-shRNA group (3 out of 133 pairs) is significantly less than that in the uninjected control group (43 out of 523 pairs) (*P*=0.01). A possible explanation is as following: in the uninjected control group, a small fraction of interneuron pairs may be coupled with rather weak electrical synapses that are beyond our electrophysiological experiment detection limit. Nonetheless, these weak electrical synapses could also promote bidirectional chemical synapse formation. However, in the Cx36-shRNA group, the suppression of Cx36 expression effectively eliminates any weak or strong electrical synapses. Therefore, the fraction of bidirectionally connected cells in the absence of electrical coupling in the Cx36-shRNA group appears to be less than that obtained in the uninjected control preparation.

Reciprocal chemical couplings between interneurons have been described in previous studies^10^,^15^,^30^. However, their physiological functions, especially in controlling synchronous activity of neocortical interneurons, are uncertain. For example, Bem *et al*.^57^ showed that reciprocal inhibitory coupling alone can only generate antiphasic firing patterns at 2–4 Hz (antisynchrony). In contrast, Hu *et al*.^55^ demonstrated that inhibitory coupling on its own can promote synchrony ∼70–80 Hz, and firing synchrony by electrical coupling was considerably less precise than synchrony by inhibitory chemical or dual connectivity^44^. Furthermore, Gibson *et al*.^43^ found that the reciprocal inhibitory postsynaptic potentials promote anti-synchronous firing among electrically coupled interneurons at frequencies <100 Hz. Given that the maximum firing frequency of layer 1 interneurons is <50 Hz, we compared the firing synchrony in the three interneuron groups (E-coupled, C-coupled and D-coupled pairs) at 40 Hz γ-frequency (30–50 Hz) and found that firing synchrony of E- and D-coupled pairs was significantly higher than that of C-coupled pairs. Moreover, synchrony driven by dual coupling was slightly lower than that driven by electrical coupling, suggesting that bidirectional inhibitory coupling promotes anti-synchronous firing among electrically coupled interneurons at 40 Hz γ-frequency. In addition, a modelling study may yield insights whether bidirectional chemical connectivity between interneurons may have significant effects on network synchrony/oscillation. Overall, our results corroborate previous findings^43^,^44^,^55^,^57^ that precise firing synchrony between cortical interneurons depend on: (1) the mode of coupling; (2) the strength of coupling; (3) the firing frequency; and (4) the subtypes of interneurons (intrinsic membrane properties).

Although our study represents the first demonstration of a link between electrical coupling and bidirectional chemical synapse formation among neocortical interneurons, mechanisms regulating the link remain largely unknown. A variety of mechanisms could account for this regulation. For example, gap junction channels allow the permeation of inorganic ions (Na^+^, K^+^, Ca^2+^ and so on) and small molecules (cAMP and IP_3_ and so on)^58^. These chemical species have been implicated as second messengers, and could play a role in the bidirectional chemical synapse formation among neocortical interneurons. Another potential cellular mechanism could be that electrical coupling in the early postnatal stage facilitates synchronized activity, which in turn promotes bidirectional chemical synapse formation, between neocortical interneurons (Hebbian theory). Indeed, we observed that electrical coupling promoted AP generation and synchronous firing between layer 1 interneurons (Fig. 10). Moreover, disruption of electrical coupling by Cx36-shRNA expression significantly reduced synchronous firing (Supplementary Fig. 13) and prevented bidirectional chemical synapse formation between layer 1 interneurons (Fig. 8). However, our results do not explain why gap junctions only promote bidirectional chemical synapse formation but have no effect on the unidirectional chemical synapses (Fig. 8h,i). It is worth noting that during early postnatal stage, GABA released by GABAergic interneurons can be excitatory to postsynaptic neurons^59^, indicating interneurons could have similar plastic and Hebbian properties as excitatory neuron at the early postnatal stage.

Remarkably, we found that a deficiency of electrical coupling increased the peak amplitude and frequency of mEPSCs in layer 1 interneuron (Fig. 9). We postulate that, lack of electrical communication between layer 1 inhibitory interneurons may lead to a reduced level of coordinated membrane depolarization (for example, APs) of layer 1 interneurons, which in turn results in a decreased inhibitory synaptic input to pyramidal neurons. A reduced inhibition of pyramidal neurons may lead to enhanced miniature EPSC frequency and amplitude.

In summary, our study not only systematically characterized the development of GABAergic and electrical connections between layer 1 interneurons but also revealed that bidirectional chemical coupling as well as electrical coupling fine-tunes interneuron firing synchrony in layer 1 of the neocortex. Moreover, we provided clear evidence of a functional role for gap junction-mediated electrical coupling in regulating precise circuit assembly of layer 1 interneurons at the individual cell resolution. The precise cellular and molecular mechanisms underlying the development of gap junction-modulated fine-scale synaptic connections of interneurons remain to be determined. It will also be interesting to explore whether the principles that we learned on layer 1 interneurons are applicable to interneurons in other cortical layers or brain regions.

## Methods

### Animals

CD-1 mice and GAD67-eGFP (Δneo) transgenic mice^60^,^61^ were used for this study. The date of birth of the mouse was defined as postnatal day 1 (P1). The advantage in using transgenic mice is the strong and robust eGFP expression in GABAergic neurons. In the neocortex, all the eGFP-positive (eGFP^+^) cells in GAD67-eGFP mice were GABAergic neurons, and ∼95% of the GABAergic neurons were eGFP-positive after birth^60^. All experiments were conducted in accordance with the guidelines for animal research and use at Fudan University.

### Plasmid and lentivirus preparation

The shRNA plasmids were generated by inserting the hairpin oligonucleotides into the FUGW-H1 lentiviral construct as previously described^62^,^63^. FUGW-H1 empty vector was a gift from Qiu Zilong lab (Addgene plasmid # 40625). Oligonucleotides containing a 21-mer sequence, followed by a loop sequence (5′- CTCAAGAGA -3′) and the reverse complement, were synthesized and cloned into the FUGW-H1 lentiviral vector using XbaI/BamHI cloning sites. The 21-mer sequences are as follows: Scrambled control: 5′- GGCACATTAGGAACCATACAT -3′ (ref. 7); Cx36-knockdown (targeting the GJD2 (connexin 36) CDS region): 5′- GGTGAATGGCATGAGTCAAAC -3′ (the candidate list of shRNA sequences which were targeted to Cx36 is in Supplementary Fig. 9a). The resulting plasmid construct is illustrated in Fig. 6a.

The lentivirus was packaged by Obio Technology Co., Ltd. In brief, the plasmids were co-transfected with pVSV-G and pCMVd8.9 into 293 T cells. Supernatant was harvested 2 and 3 days later and further concentrated by ultracentrifugation. Viral titres were measured by serial dilution in 293 T cells followed by flow cytometric analysis after 48 h. Lentiviruses were used to infect cells at a multiplicity of infection of 10, and added to primary cultured cortical cells 12 h after plating (Supplementary Fig. 9b).

### *In vivo* lentivirus injection

We used a self-made glass syringe to inject the lentivirus with fast green (2.5 mg ml^−1^, Sigma) with the needle held parallel to the cortical surface (Fig. 6e), precisely into the gap between dura and cortical layer 1 of CD-1 mice at P1. The syringe consisted of a tiny needle (removed from sterile insulin syringes, BD, REF328420) and a glass tube (PCR micropipets, Drummond Scientific, 5-000-1001-X10), glued by epoxy resin. The needle was used to ensure a minimal invasive effect. This protocol allows the virus to spread to a larger area of layer 1 than other injection methods. Fast green enabled the visualization of the extent of diffusion of virus (Fig. 6f). There were four injection sites in the brain: posterior 1 mm from bregma; anterior 1 mm from lambda; 2 mm lateral to the midline in each brain hemisphere; and ∼1 μl virus solution was injected at each site.

### Primary neuronal cultures

The cortical region was dissected from embryonic day 18 (E18) embryos. The intended brain regions were dissected and transferred into chilled dissecting medium (Hank's balanced salt solution, Gibco), then digested for 30 min at 37 °C in Neurobasal medium (Gibco) containing papain (20 μl ml^−1^) and DNase I (100 μg ml^−1^). Approximately 3 × 10^6^ cells per well in a six-well plate were plated in 1.5 ml per well Neurobasal medium, supplemented with 2% B27 (Invitrogen), 1% Glutamax (Gibco) and 1% Penicillin-Streptomycin (Gibco) on poly-D-lysine-coated plates. Cells were fed every 2 days *in vitro* (DIV) and transduced 24 h after plating. Cells were maintained for a further 4–5 DIV then harvested for western blot and quantitative real-time PCR analysis.

### Western blot

Protein was isolated using 2% SDS with EDTA from virus-infected cells, and protein concentration was calculated using the BCA assay (Pierce). Equal amounts of protein (15–20 μg per lane) were loaded onto 12% bis-tris-polyacrylamide gels (Beyotime) and transferred onto polyvinylidene fluoride membranes (Millipore). Membranes were blocked with 5% non-fat milk (Applichem) for 2 h at room temperature (RT) and blotted overnight with antibody at 4 °C: Cx36 (1:250, Invitrogen, catalog no. 51-6300) and tubulin (1:2,000, Sigma, catalog no. T6793). Horseradish peroxidase-conjugated secondary antibodies (Jackson ImmunoResearch) were used at 1:10,000 for 1 h at RT. After treatment with enhanced chemiluminescence (ECL) (Thermo) chemical substrate, the membrane was exposed and analysed with a gel imaging system (Bio-Rad Gel Doc XR+). Images were cropped for presentation. Full-size images are presented in Supplementary Fig. 14.

### Quantitative real-time PCR analysis

Total RNA was extracted from virus-infected cells using Trizol reagent (Takara, RNAiso plus). cDNA was prepared with SuperScript II First-Strand Synthesis System (Invitrogen) and used as template for PCR. PCR core reagents and SYBR green (TOYOBO) were used with 200 nm of forward and reverse primers. Real-time quantitative PCR was performed with the Eppendorf Mastercycler Pro system. A standard curve was generated for each primer pair, and genes of interest were assigned a relative expression value extrapolated from this standard curve using the threshold cycle (Ct) according to Eppendorf instructions. All expression values were normalized against GAPDH. All amplifications were done in duplicate, and at least three technical and three biological replicates were performed. PCR primer sequences are as follows: Cx36, forward, 5′- CCAATTTCGGCAGGACTCAG -3′, reverse, 5′- TAGACCTTCACAGCGAGCATC -3′; GAPDH, forward, 5′- CATGGCCTTCCGTGTTCCTA -3′, reverse, 5′- CCTGCTTCACCACCTTCTTGAT -3′.

### Immunohistochemistry

Mice at various postnatal ages (P1-25) were perfused intracardially with cold PBS (pH 7.4) and 4% paraformaldehyde (PFA) in PBS (pH 7.4). Brains were removed from the skull, postfixed overnight, washed in PBS and sectioned coronally at 60 μm on a vibratome (Leica VT1000S). Sections were incubated with the primary antibodies in PBS containing 1% bovine serum albumin, 0.5% Triton X-100 and 0.05% sodium azide for 36–48 h at 4 °C. After washing in PBST (0.1% Triton X-100 in PBS) five times for 50 min, sections were incubated in fluorescent secondary antibodies overnight at 4 °C and subsequently washed five times in PBS for 50 min. Mounted slices were visualized under fluorescent illumination with the BX41 microscope (Olympus, Japan). The following primary antibodies were used: rabbit anti-Connexin 36 (Cx36, 1:500, Invitrogen #51-6300); goat anti-calretinin (CR, 1:1,000, Millipore #AB1550); mouse anti-Reelin (1:1,000, Millipore #MAB5364); rabbit anti-neuropeptide Y (NPY, 1:500, Immunostar #22940); chicken anti-eGFP (1:1,000, Aves #1020); rabbit anti-vasoactive intestinal peptide (VIP, 1:400, Immunostar #22700); goat anti-somatostatin (SOM, 1:250, Santa cruz #sc-7819); and rabbit anti-GABA_A_Rδ (1:100, Phosphosolution #868-GDN). The secondary antibodies were: donkey anti-goat (conjugated to Alexa Fluor 488 and Alexa Fluor 555, 1:500, Invitrogen); donkey anti-rabbit (Alexa Fluor 488 and Alexa Fluor 555, 1:500, Invitrogen); donkey anti-mouse (Alexa Fluor 488, Alexa Fluor 555 and Alexa Fluor 647, 1:500, Invitrogen); and donkey anti-chicken (DyLight 488, 1:500, Jackson immunoResearch). The chicken anti-eGFP and donkey anti-chicken DyLight 488, which are IgY, while the remaining antibodies are IgG. Confocal images were obtained by Olympus FV1000 confocal microscope with a × 40, × 60 and × 100 objective and 1 μm (for Fig. 6g and Supplementary Fig. 2a) or 0.2 μm (for Supplementary Figs 6b and 8a) z-step size. Digital images were brightness, contrast and colour balanced by adjusting in Photoshop CS5 (Adobe Systems). The laminar borders of layer 1 were distinguished based on 4′, 6-diamidino-2-phenylindole staining.

### Slice preparation, electrophysiology and data analysis

P1-5 GAD67-eGFP transgenic mice and P6-25 CD-1 WT mice were anaesthetized by 1% isoflurane and 0.5–1.0 l min^−1^ oxygen. Their brains were then dissected from the skulls. For mice up to P5 (including P5), the somatosensory cortex was whole mounted. From P6-12, horizontal sections of the somatosensory cortex were cut at a thickness of 200 μm with a vibratome (Leica VT1000S) in a solution containing (mM) 120 choline chloride, 2.6 KCl, 0.5 CaCl_2_, 7 MgCl_2_, 26 NaHCO_3_, 1.25 NaH_2_PO_4_, 15 D-glucose and 1.3 ascorbic acid, then incubated in artificial cerebrospinal fluid (ACSF) containing (mM) 126 NaCl, 3 KCl, 1.25 KH_2_PO_4_, 1.3 MgSO_4_, 3.2 CaCl_2_, 26 NaHCO_3_ and 10 glucose (pH adjusted to 7.3–7.4), bubbled with 95% O_2_/5% CO_2_. From P13-25, a modified protective slicing and recovery method was used to improve the health of the slices from aged animals^64^. In brief, mice were deeply anaesthetized with isofluorane and transcardially perfused with 10 ml of chilled oxygenated (95% O_2_, 5% CO_2_) NMDG-HEPES Recovery Solution (NMDG cutting Solution) containing (mM) 93 *N*-methyl-D-glucamine (NMDG), 2.5 KCl, 1.2 NaH_2_PO_4_, 30 NaHCO_3_, 20N-2-hydroxyethylpiperazine-N'-2-ethanesulfonic acid (HEPES), 25 D-glucose, 5 sodium ascorbate, 2 thiourea, 3 sodium pyruvate, 10 MgSO_4_.7H_2_O, 0.5 CaCl_2_.2H_2_O and 12 NAC (pH adjusted to 7.3–7.4). Brains were quickly removed, cut into 200 μm horizontal slices with the Compresstome VF-300 microtome (Precision Instruments, Inc.) in chilled oxygenated (95% O2, 5% CO2) NMDG cutting solution and then incubated in the same solution for 10 min at 34.5 °C, followed by recovery for 1 h at RT in modified HEPES with ACSF solution (modified-ACSF) containing (mM) 94 NaCl, 2.5 KCl, 1.2 NaH_2_PO_4_, 30 NaHCO_3_, 20 HEPES, 25 D-glucose, 5 sodium ascorbate, 2 thiourea, 3 sodium pyruvate, 2 MgSO_4_.7H_2_O, 2 CaCl_2_.2H_2_O and 6 NAC (pH adjusted to 7.3–7.4). After 1 h, the slices were moved into normal ACSF solution at RT, bubbled with 95% O_2_/5% CO_2_. The schema of slice preparation is shown in Supplementary Fig. 1. The cutting angle was consistent in all experiments.

Whole-mounted and horizontal slices ensured a sufficient preservation of both layer 1 cells' axonal and dendritic arborizations. Individual cells were identified with an infrared-DIC microscope BX51WI (Olympus) equipped with epifluorescence illumination, water immersion objective (× 20 and × 60) and an ORCA-R2 CCD camera (Hamamatsu). Furthermore, the distance at *Z* axis between recorded cells is ∼0–2 somata size (<30 μm). Glass recording electrodes (8–12 MΩ resistance) were filled with an intracellular solution containing (in mM): 93K-gluconate, 16 KCl, 2 MgCl_2_, 0.2 EGTA, 10 HEPES, 2.5 ATP-Mg, 0.5 GTP-Na_2_, 10 Na-phosphocreatine, 0.5% Alexa Fluor 568 hydrazide, 0.4% neurobiotin, pH 7.25 and 290–300 mOsm. Recordings were collected and analysed using two Axon Multiclamp 700B amplifiers, Digidata 1440A (Molecular Devices) and pCLAMP10 software (Molecular Devices). Slices were transferred to a recording chamber perfused with warm bubbled ACSF at 32–34 °C and quadruple whole-cell recordings were performed in all pairs. In some cases, two or three neurons were successfully patched or survived. These pairs were included in quantitative analysis. During recording, series resistance was continuously monitored. Recordings with series resistance of >30 MΩ were excluded from analysis. Considering the high risk of damage of the recording induced by the oscillation of Rs compensation, particularly during long recordings, we did not compensate the series resistance. After establishing the whole-cell configuration, accumulated fine depolarizing currents (3 pA) were injected into the neuron to induce steady-state AP from resting membrane potential, which can reflect the intrinsic electrophysiological properties of the interneurons. AP threshold was determined as the voltage at which the upslope velocity of the AP increased rapidly. The membrane voltages were plotted against their first-time derivative (*d*V/*d*t, phase–plane plot) using Clampfit 10 software (Molecular Devices). AP threshold was selected as the voltage at which *d*V/*d*t exceeded three times the s.d. of all the preceding data points^65^,^66^. AP amplitude was measured from the threshold to the peak of AP. AP width was measured as the duration between its half-amplitude that reflects AP duration. In quadruple recordings, synaptic connections were assessed by sequential injections of trains of depolarizing or hyperpolarizing currents to one neuron to evoke APs or hyperpolarization, and monitoring postsynaptic responses of the remaining neurons maintained at −70 mV. For every possible pair, connections were tested in both directions in at least 20 trials, with single APs being generated in each presynaptic neuron. uIPSC and electrically coupled response were further determined by averaging 20 trials. Electrical coupling efficiency was assessed by injecting currents to trigger hyperpolarization or depolarization under current-clamp mode in at least 10 trials, and measuring the voltage change in the postsynaptic neurons. Spontaneous activity was recorded, while neurons were maintained at −70 mV.

For mEPSC and mIPSC recordings, we used modified intracellular solution to adjust the reversal potential of the GABA-A receptor response (127.5 mM caesium methanesulfonate, 7.5 mM CsCl, 10 mM HEPES, 2.5 mM MgCl_2_, 4 mM Na_2_ATP, 0.4 mM Na_3_GTP, 10 mM sodium phosphocreatine, 0.6 mM EGTA, pH 7.25). Under our recording conditions, miniature EPSCs and IPSCs were recorded in the same cells for 3 min by voltage-clamping the membrane potential at the reversal potential of the GABAergic current (−60 mV) and glutamatergic current (+10 mV), respectively. Bath application of an a-amino-3-hydroxy-5-methyl-4-isoxazolepropionic acid receptor blocker (NBQX, 10 μM) and *N*-methyl-D-aspartate receptor blocker (D-APV, 50 μM) or GABA-A receptor blocker (bicuculline, Sigma-Aldrich, #14340, 10 μM) completely blocked the mEPSC (at −60 mV) or mIPSC (at +10 mV) events, respectively. All experiments were performed in the presence of tetrodotoxin (TTX, 5 μM). Spontaneous and miniature PSCs (sPSCs and mPSCs) were analysed using MiniAnalysis 6.0 Program (Synaptosoft, Inc). sPSCs and mPSCs had a fast (<3 ms) rise time and exponential decay. A value of twice the root mean squared noise level for a given recording was used as the detection limit for synaptic current peak amplitude. After an automated search, undetected and false positives were corrected by visual inspection.

After the recordings were completed slices were fixed in 4% PFA in PBS (pH 7.4) and the morphology of recorded neurons loaded with Alexa Fluor 568 hydrazide through the recording pipette was visualized using an Olympus FV1000 confocal laser scanning microscope. Z-series images were taken at 1–3 μm steps and analysed using FluoView (Olympus), Neurolucida (MicroBrightField, Inc.) and Photoshop (Adobe Systems). In some recording experiments, neurobiotin was later visualized with streptavidin-conjugated Alexa Fluor 568 (Invitrogen).

Normalized cross-correlograms of firing patterns were analysed as previously described^67^. In brief, the number of neuron 1 s that fired within a time interval [*n*Δ*t*, (*n*+1)Δ*t*] from spikes fired by neuron 2 was calculated (*y*_*n*_=counts/bin; bin width, Δ*t*=1 ms). The cross-correlogram *y*_*n*_ was normalized in standard scores [*Z*=(*y*_*n*_−γ_E_)/*s*_*y*_, with *γ*_E_=f_1_*f_2_*T*Δt (f_1,2_, average firing rate of neurons 1 and 2; T, recording time) and *s*_*y*_, s.d. of *y*_*n*_]. Peaks in the cross-correlogram were considered significant if individual bins exceeded expected value by 3 s.d. (*Z*-score>3).

Synchrony of spike firing between two layer 1 neurons was quantified using the JBSI as previously reported^45^. In brief, we placed a synchrony time window (set as±*S*, *S*=2 ms) in the centre of each spikes from the faster spike train, and the number of spikes (set as *SYN*(S)) from the slower spike train occurring within the time windows was counted. The jitter window (set as±*J*, *J*=2*S*) was then put in the centre of each spikes of the slower spike train (the number of spikes set as *N*), and the probability that a jittered spike would fall into a synchrony window was further calculated. The number of coincidences expected after a random jitter (±*J*), <*SYN*^J^(*S*)>, is set as the sum of all these probabilities. The normalized JBSI is: 2[*SYN*(*S*)−<*SYN*^J^(*S*)>]/*N*.

### Morphological reconstruction and quantification

Layer 1 neurons were filled with 0.5% Lucifer yellow by the patch pipette. Subsequently, brain slices were fixed in 0.1 M PBS (4% PFA) overnight. The primary and secondary antibodies used to visualize the labelled cells were anti-Lucifer yellow (1:2,000, Invitrogen, #A5750, rabbit IgG) and anti-rabbit IgG (Alexa Fluor 488, 1:250, Invitrogen), respectively. A large region Z-stack images were obtained on the spinning disk confocal system (PerkinElmer UltraView system) with mosaic acquisition mode. Successfully stained neurons were then reconstructed using Neurolucida (MicroBrightField).

### Data acquisition and analysis

All data are presented as mean±s.e.m., unless otherwise stated. All statistical analyses were performed using SigmaPlot 12.0 (Systat Software) and SPSS 22 (IBM). The statistical differences between groups were determined by two-tailed Student's paired *t*-test, Mann–Whitney rank sum test, χ^2^-test and Fisher's exact test, and *P* values of <0.05 were considered to be statistically significant.

### Data availability

All data supporting the findings of this study are available within the article, its Supplementary Information files, or from the corresponding authors on request.

## Additional information

**How to cite this article:** Yao, X-H. *et al*. Electrical coupling regulates layer 1 interneuron microcircuit formation in the neocortex. *Nat. Commun.* 7:12229 doi: 10.1038/ncomms12229 (2016).

## Supplementary Material

Supplementary Information Supplementary Figures 1-14 and Supplementary Tables 1-2
