# Supplementary material for: Electrical coupling regulates layer 1 interneuron microcircuit formation in the neocortex
Source: Nat Commun. 2016 Aug 11;7:12229. doi: 10.1038/ncomms12229 (PMC4987578; doi:10.1038/ncomms12229)
Supplement: Supplementary Information — Supplementary Figures 1-14 and Supplementary Tables 1-2 [file ncomms12229-s1.pdf]

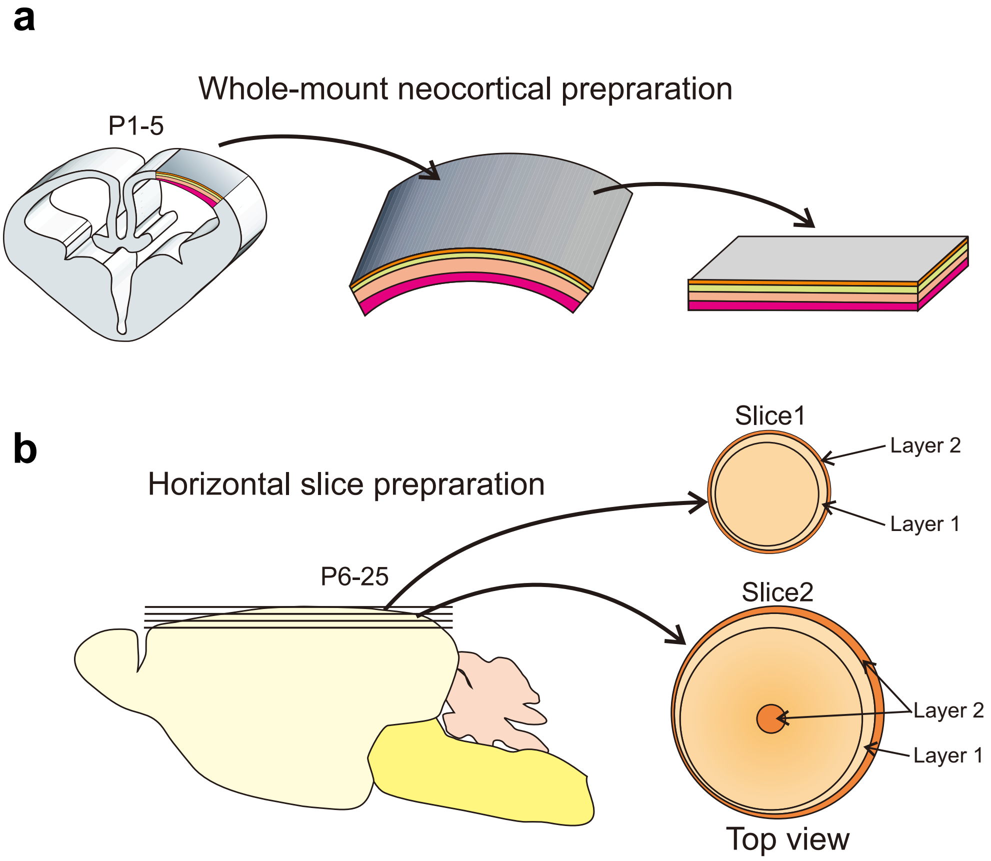


**Supplementary Figure 1. The schema of brain slice preparations.** (**a**) Whole-mount neocortical preparation from GAD67-eGFP transgenic mice aged P1-P5. (**b**) Acute horizontal slice preparation from CD-1 mice at P6-25. Only the first few slices were used for recording.

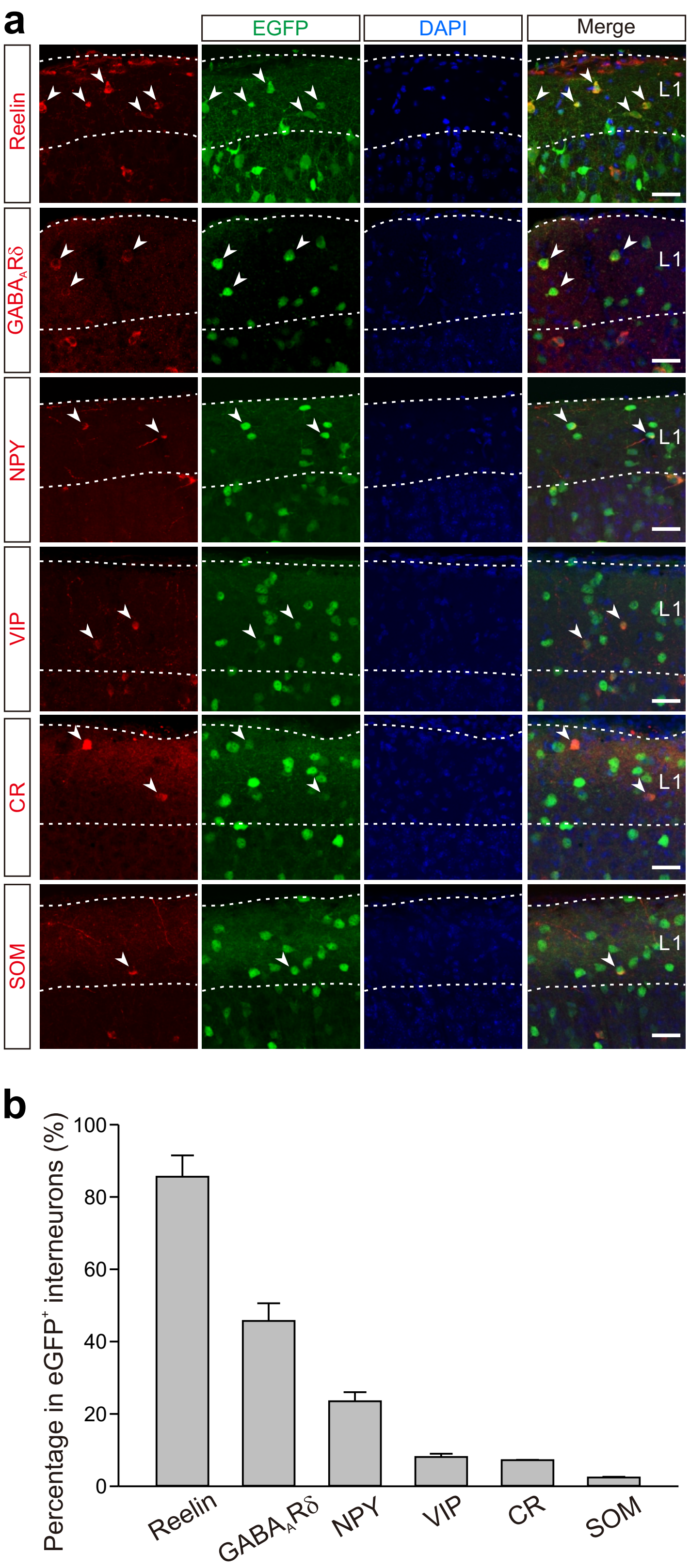


**Supplementary Figure 2. The expression of molecular markers in layer 1 interneurons. (a)** Confocal fluorescence images of coronal sections through the somatosensory cortex of GAD67-GFP mice at P20. The majority of $GFP^+$ interneurons in layer 1 expressed Reelin, $GABA_AR\delta$, NPY, VIP, CR and SOM. NPY, neuropeptide Y; VIP, vasoactive intestinal polypeptide; CR, calretinin; SOM, somatostatin. Scale bar: 20 μm. (**b**) Quantification of the percentage of layer 1 cells immunostained for the various interneuron markers relative to the total $GFP^+$ interneurons (n = 3-5 mice per marker). Error bars represent mean ± s.e.m.

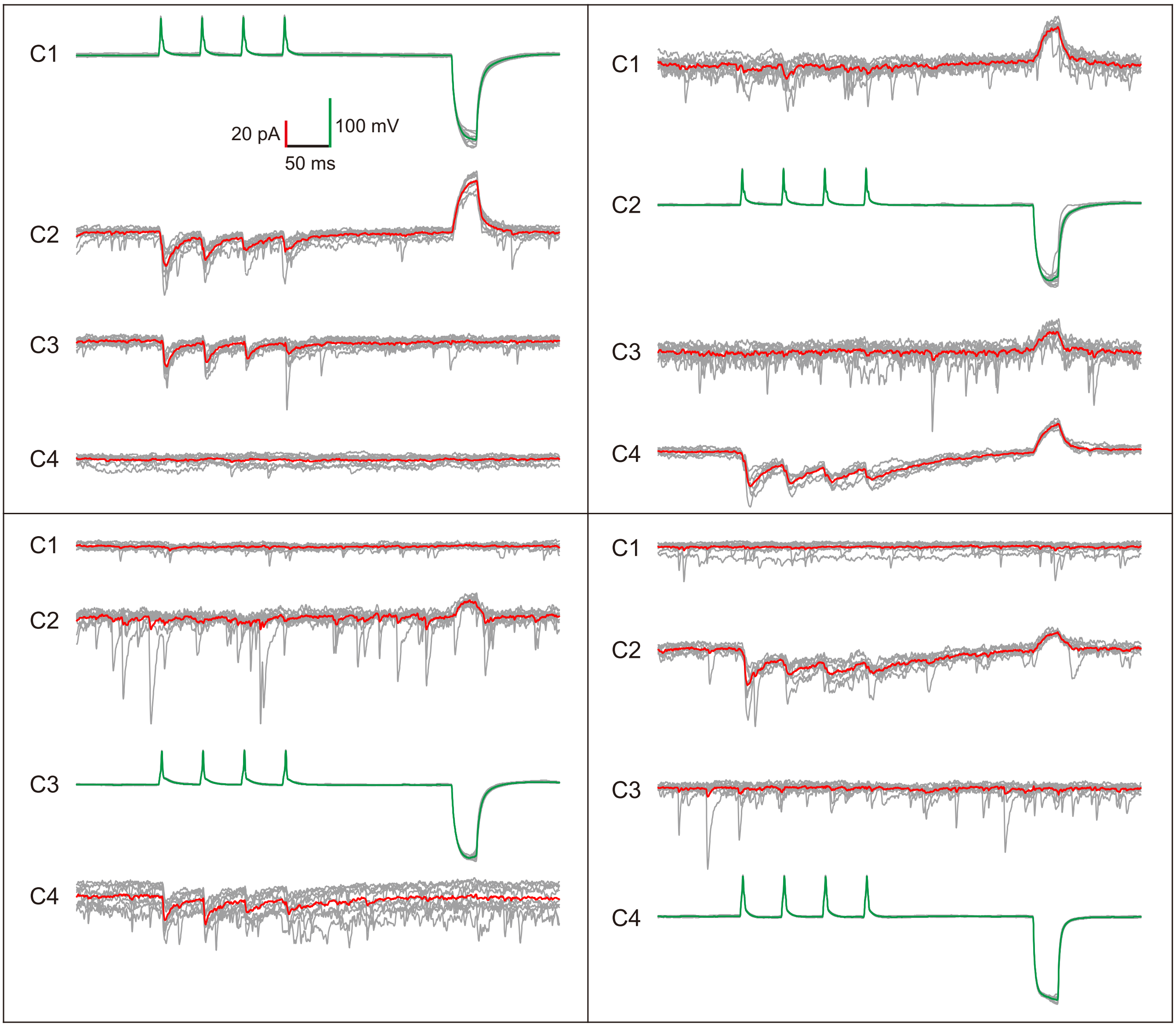


**Supplementary Figure 3. The representative traces of quadruple whole-cell recordings (related to Fig. 1e).** The sample traces of serial action potentials and hyperpolarization sequentially triggered in the presynaptic neurons (green traces) and responses (red traces) recorded in the postsynaptic neurons. The bold traces represent the average and the grey traces represent the individual recordings.

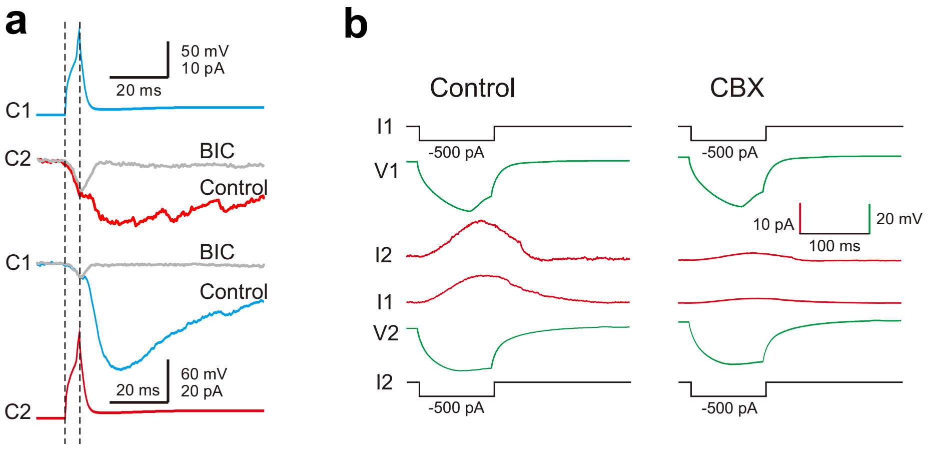


**Supplementary Figure 4. Identification of GABA-receptor-mediated and electrical coupling-mediated responses.** (**a**) Bicuculline treatment (BIC, 10 μM) remarkably reduced the amplitudes of inward currents and eliminated the responses with slow decay time. (**b**) Carbenoxolone treatment (CBX, 100 μM) abolished hyperpolarization-induced outward currents.

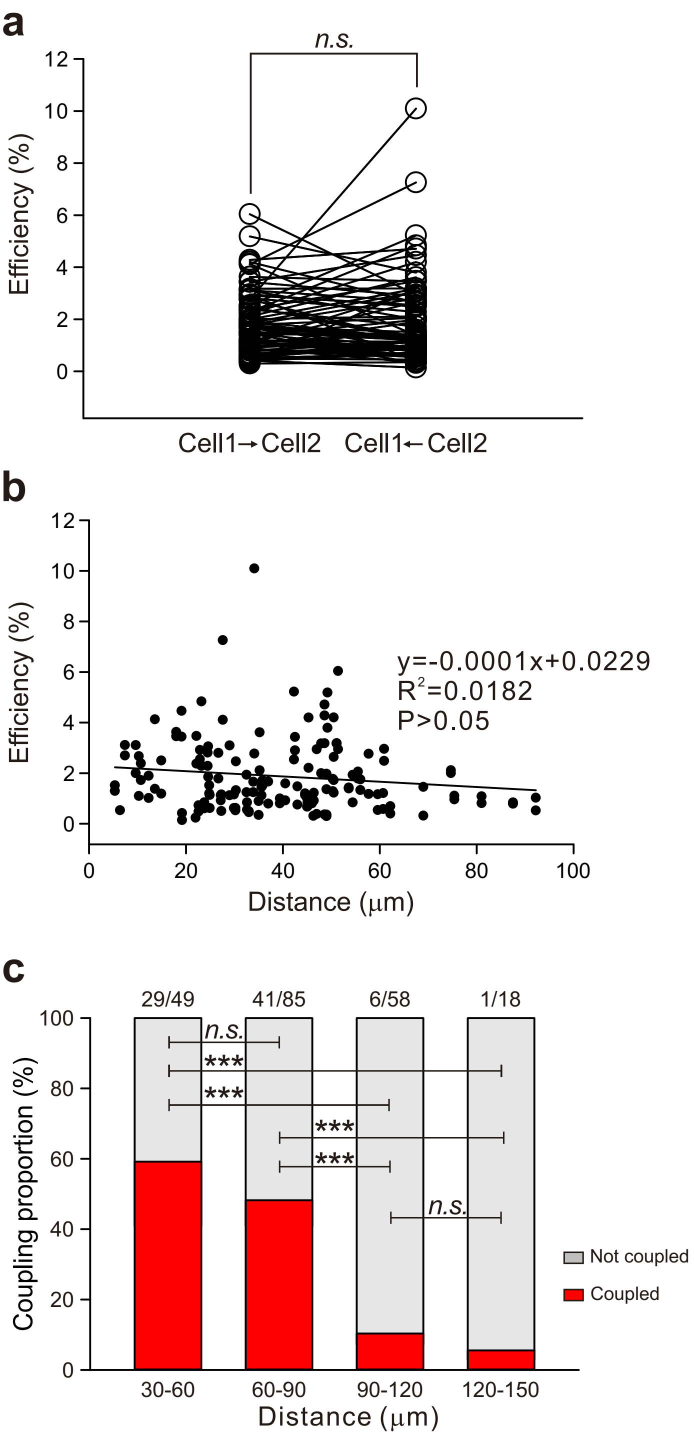


**Supplementary Figure 5. Characterization of electrical coupling between neocortical layer 1 interneurons.** (**a**) Electrical coupling was bidirectional. The bidirectional coupling coefficients of each coupled pair estimated by injecting current to either cell 1 or cell 2 were not significantly different. (**b**) The plot of coupling coefficients revealed that they are independent of the distance between somata of layer 1 interneurons ($R^2$=0.0182, $P$>0.05). (**c**) The number of electrical connections is significantly reduced with increased distance between somata. ***$P$<0.001, *n.s.*, $P$>0.05, not significant. $\chi^2$ test, Fisher's exact test, two-tailed paired $t$ test and Spearman Rank Order Correlation.

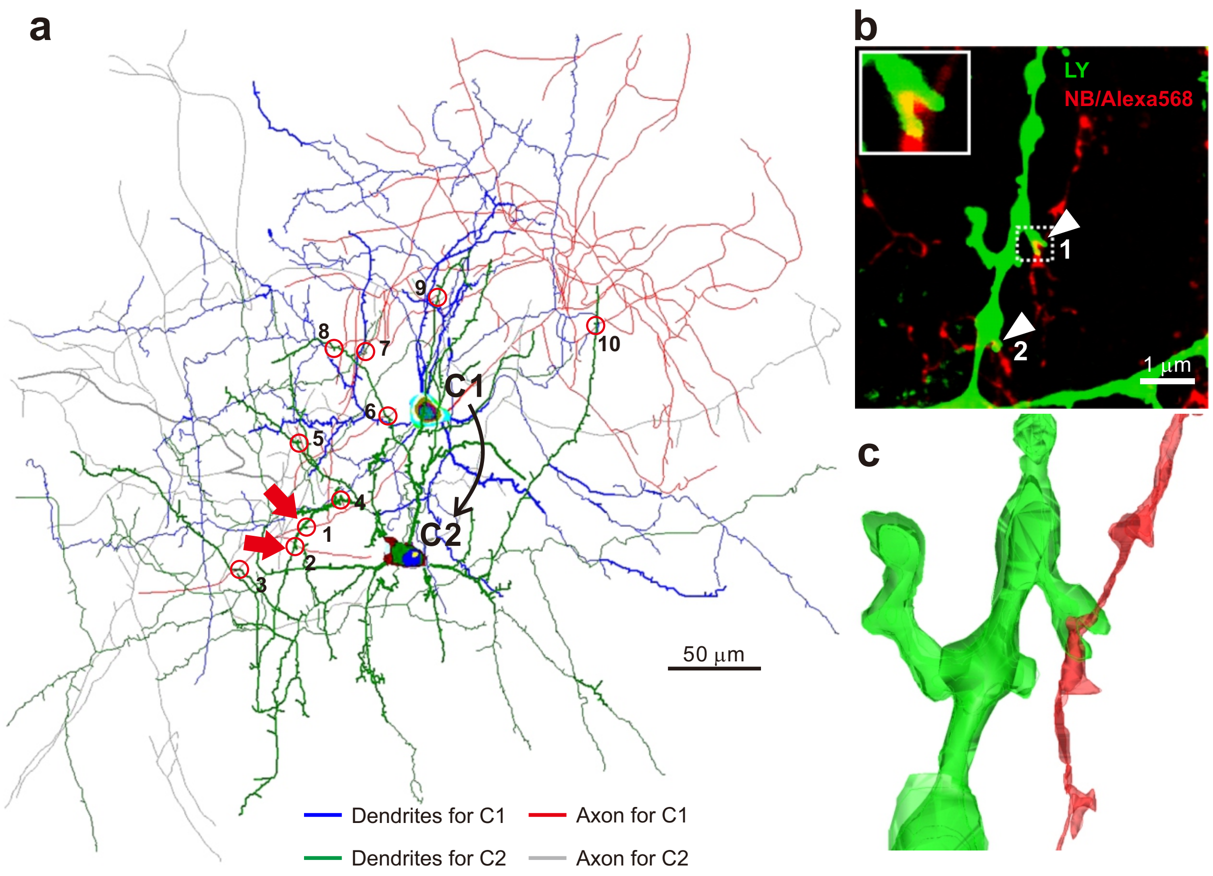


**Supplementary Figure 6. Potential points of contact of chemical synapses between two layer 1 interneurons**. (**a**) Three-dimensional reconstruction of two LS interneurons in a dual patch-clamp recording at P15. The black arrow indicates the direction of unidirectional chemical connections between cell1 (C1) and cell2 (C2). To separate the processes of the two neurons, one neuron was filled with neurobiotin (NB) and visualized with streptavidin-conjugated Alexa Fluor 568; the other neuron was filled with Lucifer yellow (LY). Red circles indicate the potential points of contact of chemical synapses. Red arrows indicate the potential points of contact #1 and #2. Note that there are 10 potential points of synaptic contact between these two interneurons. (**b**) The confocal fluorescent image of the potential synaptic contacts #1 and #2 (white arrowheads) indicated by red arrows in (**a**). Inset: high magnification view of point of contact #1. (**c**) Three-dimensional reconstruction of point of contact #1.

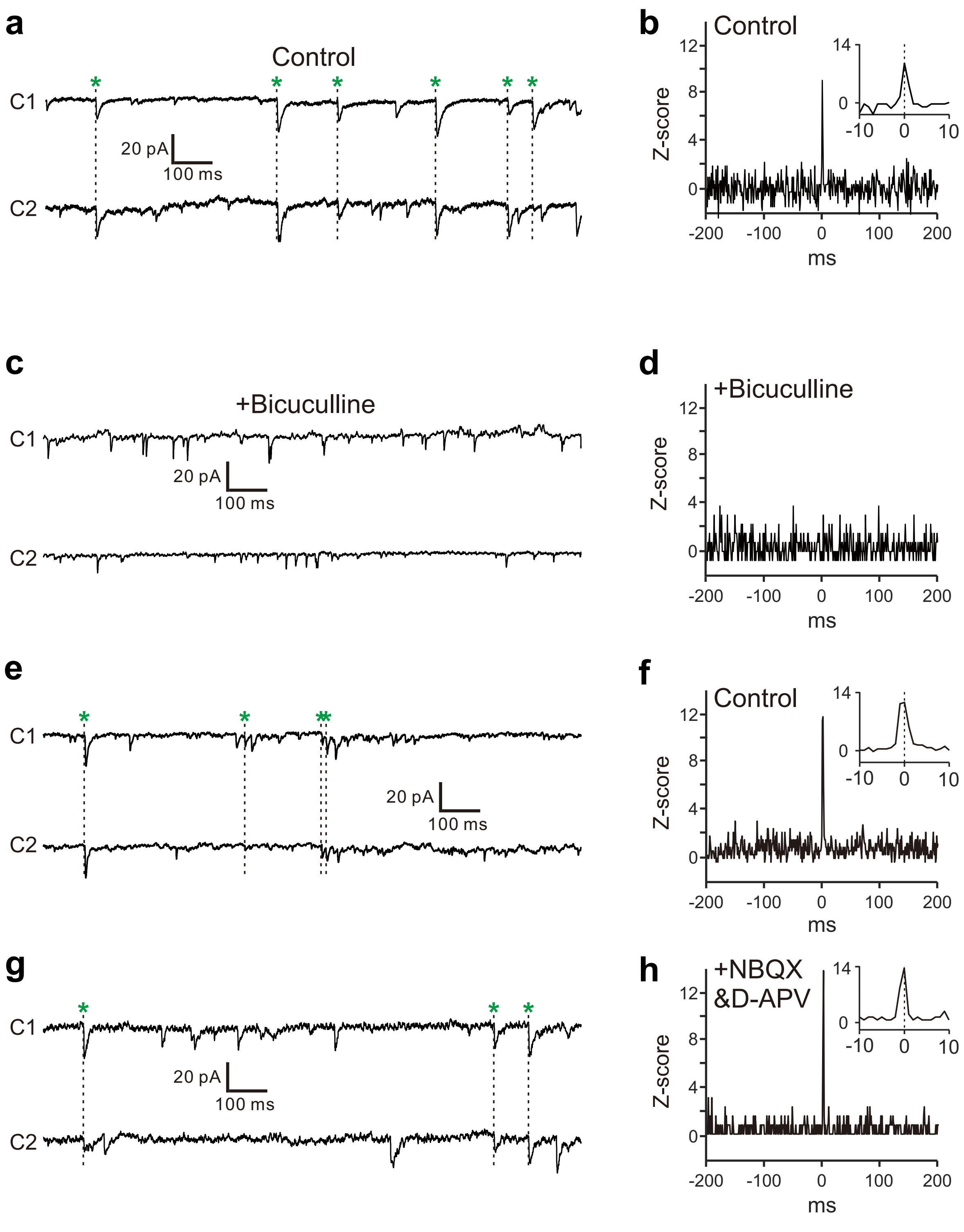


**Supplementary Figure 7**. **Synchronized spontaneous activity is mediated by GABA-A receptor.** (**a, c**) Sample traces of the spontaneous activity of two layer 1 interneurons before (**a**) and after (**c**) bicuculline treatment. Green asterisks indicate synchronized events. (**b, d**) Normalized cross-correlogram before (**b**) and after (**d**) bicuculline treatment. (**e, g**) Sample traces of the spontaneous activity of two layer 1 interneurons before (**e**) and after (**g**) NBQX and D-APV treatment. Green asterisks indicate synchronized events. (**f, h**) Normalized cross-correlogram before (**f**) and after (**h**) NBQX and D-APV treatment.

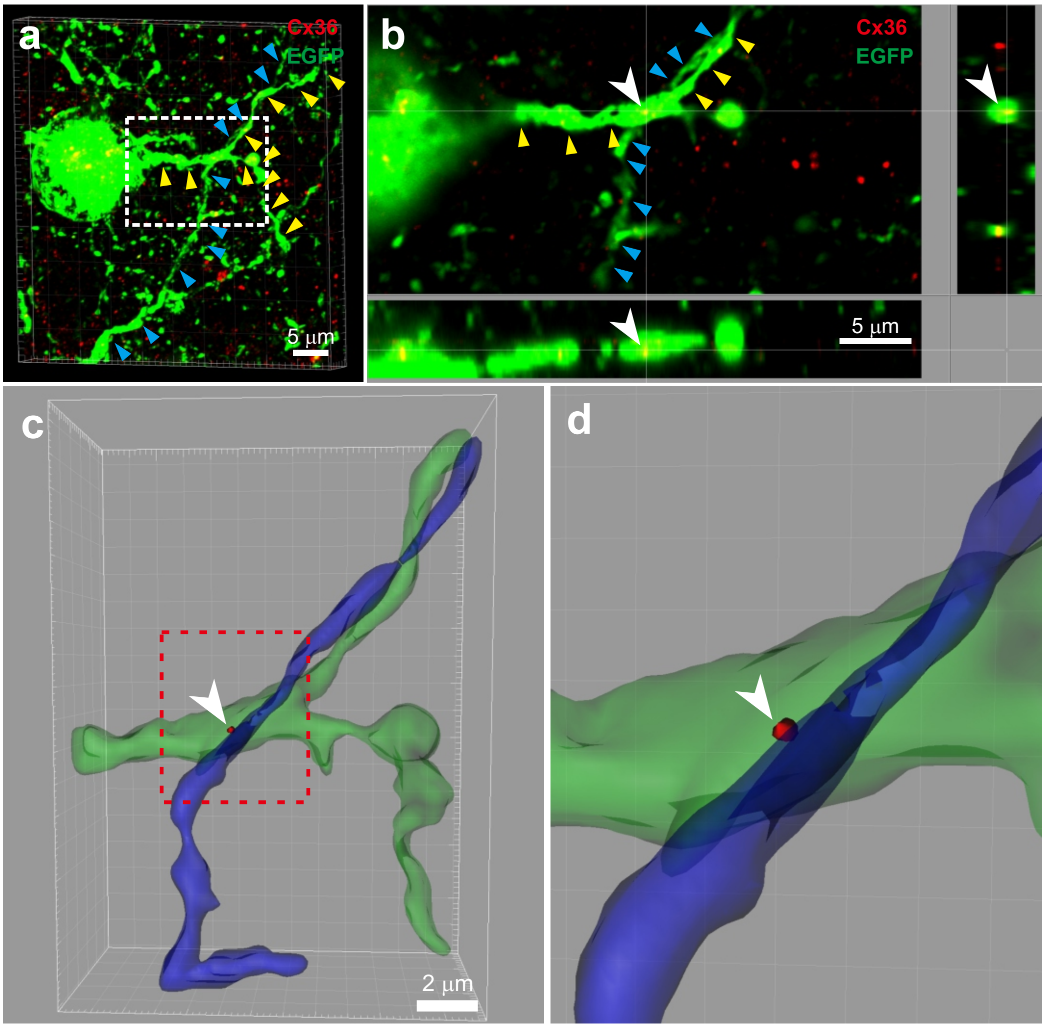


**Supplementary Figure 8. The localization of Cx36 puncta between layer 1 interneurons.** (**a**) Immunostained images of two eGFP⁺ interneurons (infected by Ctrl-shRNA virus) in layer 1 with antibody specific for Cx36 (red). Yellow and blue arrowheads indicate the dendrites from two layer 1 interneurons. (**b-d**) The presence of Cx36 puncta (indicated by white arrowheads) at the dendrodendritic contact of these two interneurons in the Z-axis cross section (**b**) magnified from white dotted rectangular region in (**a**), in the three-dimensional reconstruction ((**c**) and (**d**), (**d**) shows a magnification of the red dotted region in (**c**)).

**a**

| No. | Cx36 shRNA target sequence | KD Efficiency |
|---|---|---|
| 1 | CAGATTGCTTAGAGGTTAA | - |
| 2 | TGATCTTCCGGATACTCATTG | - |
| 3 | GGACGCACATAGATTGCTT | + |
| 4 | GCAGCACTCCACTATGATT | - |
| 5 | GCTCCGAAGACAGGAAGGT | +/- |
| 6 | CCATCTGGGATGGCGGAAGAT | +/- |
| 7 | GACAGTCTTTCTGGTGTTCAT | +/- |
| 8 | GGTGAATGGCATGAGTCAAAC | +++ |

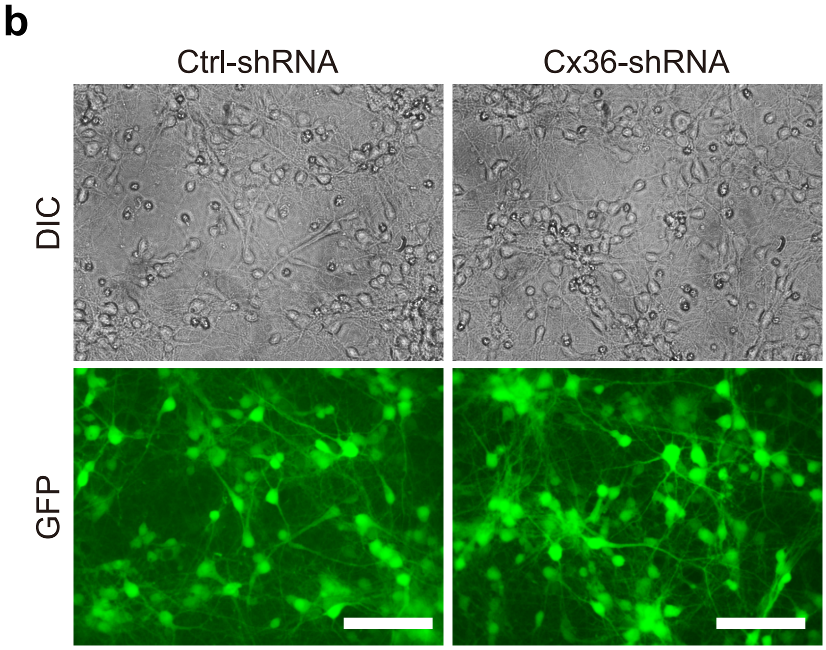


**Supplementary Figure 9. The knockdown efficiency of Cx36-shRNA lentiviral constructs.** (**a**) The list of candidate shRNA sequences targeted to connexin 36. + represents small knockdown (KD) efficiency, - represents no KD, +/- represents uncertain KD efficiency and +++ represents the highest efficiency. (**b**) DIC and fluorescence micrographs of cultured primary cortical neurons from E18 mice 96h after infection with Ctrl-shRNA or Cx36-shRNA lentiviruses. Scale bar, 100 µm.

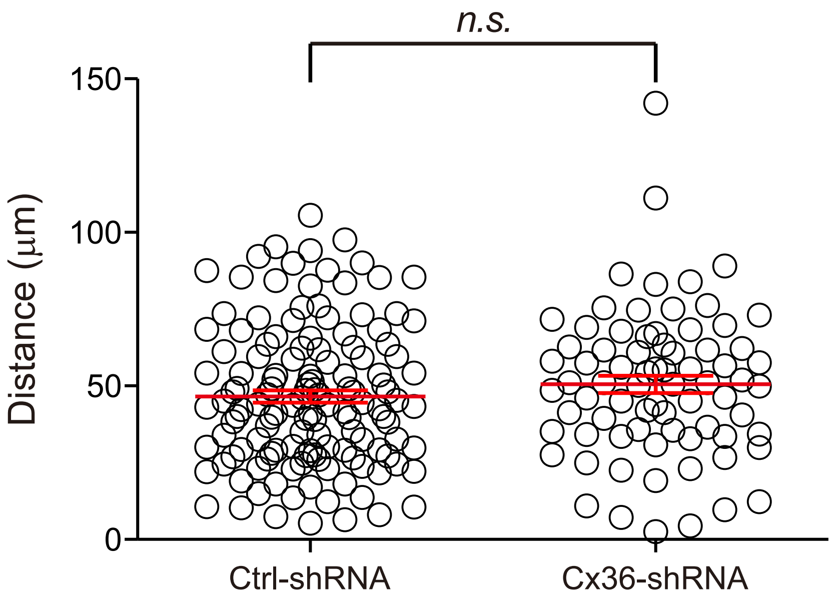


**Supplementary Figure 10. The distance between neurons recorded in Ctrl-shRNA and Cx36-shRNA groups.** There was no significant difference in the distances between recorded eGFP$^+$ neurons in Ctrl-shRNA and Cx36-shRNA groups. n = 75 pairs from 21 mice for Ctrl-shRNA group, 134 pairs from 37 mice for Cx36-shRNA group. *n.s.*, *P*>0.05, not significant. Mann-Whitney rank sum test. Error bars represent mean ± s.e.m.

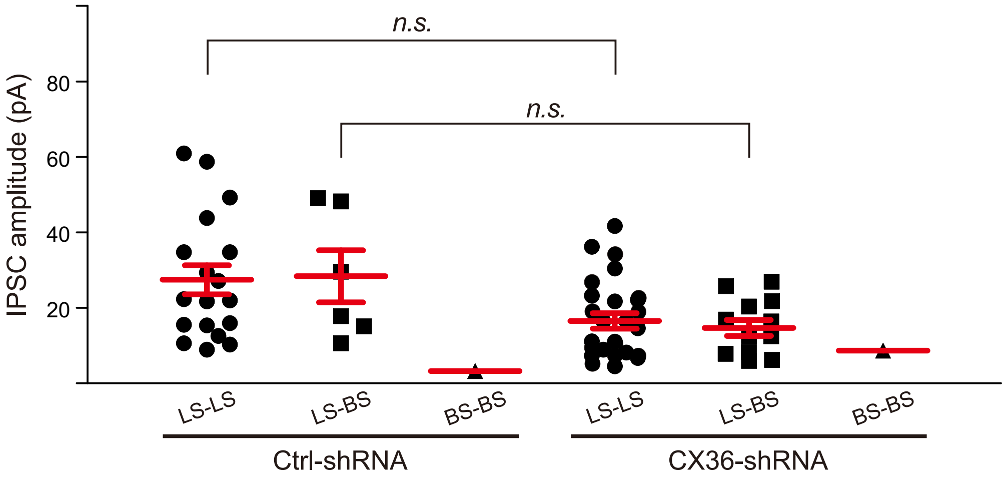


**Supplementary Fgure 11. The peak amplitude of unitary IPSCs between layer 1 interneurons.** The peak amplitude of uIPSCs was not significantly different between Cx36-shRNA neurons (16.98 ± 2.03 pA in LS-LS pairs, n = 27; 15.09 ± 2.13 pA in LS-BS pairs, n = 13; 8.71 pA in BS-BS pairs, n = 1) and Ctrl-shRNA neurons (26.28 ± 3.88 pA in LS-LS pairs, n = 18; 28.41 ± 6.70 pA in LS-BS pairs, n = 6; 3.26 pA in BS-BS pairs, n = 1). *n.s.*, $P>0.05$, not significant. Mann-Whitney rank sum test. Error bars represent mean ± s.e.m.

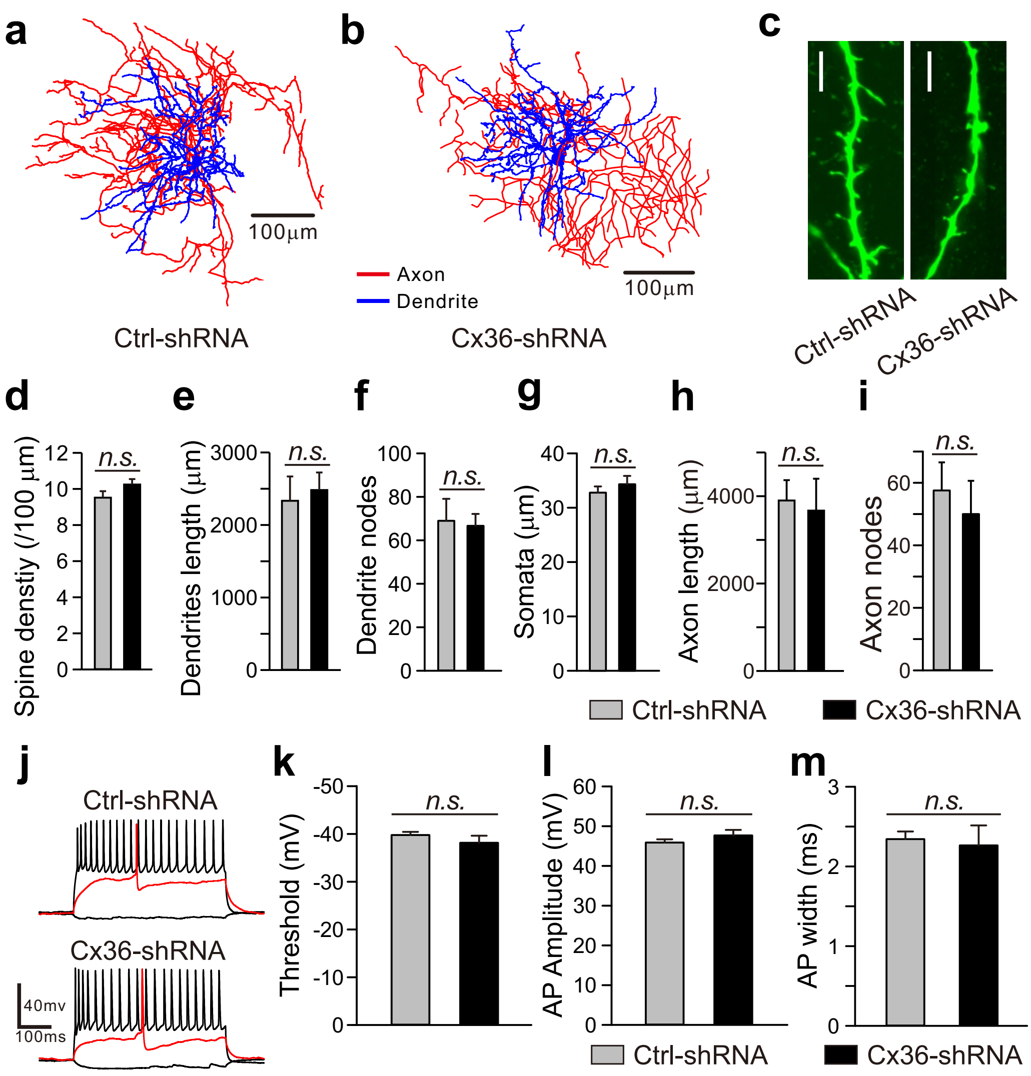


**Supplementary Figure 12. The interneurons expressing Cx36-shRNA exhibited normal morphological and intrinsic electrophysiological properties.** (**a, b**) Morphological reconstructions of layer 1 interneurons in Ctrl-shRNA group (**a**) and Cx36-shRNA group (**b**). (**c**) Representative graphs of dendritic spines in Ctrl-shRNA neuron (left) and Cx36-shRNA neuron (right). Scale bars, 5 μm (left), 10 μm (right). (**d**) Quantitative analysis of spine density. (**e-i**) Quantitative analysis of dendritic length (**e**) dendritic nodes (**f**) the maximum somata diameter (**g**) axonal length (**h**) and axonal nodes (**i**) indicated no significant difference between Ctrl-shRNA neurons and Cx36-shRNA neurons (n = 19 cells from 4 mice for Ctrl-shRNA group, 15 cells from 4 mice for Cx36-shRNA group). (**j**) The sample traces of voltage responses to current injections in Ctrl-shRNA LS neurons and Cx36-shRNA LS neurons. (**k-m**) Quantitative analysis of AP threshold (**k**), AP amplitude (**l**) and AP spike width (**m**) showed no significant difference between Ctrl-shRNA LS neurons and Cx36-shRNA LS neurons (n = 45 cells from 11 mice for Ctrl-shRNA group, 25 cells from 6 mice for Cx36-shRNA group). *n.s.*, $P>0.05$, not significant. Mann-Whitney rank sum test and two-tailed paired *t* test. Error bars represent mean ± s.e.m.

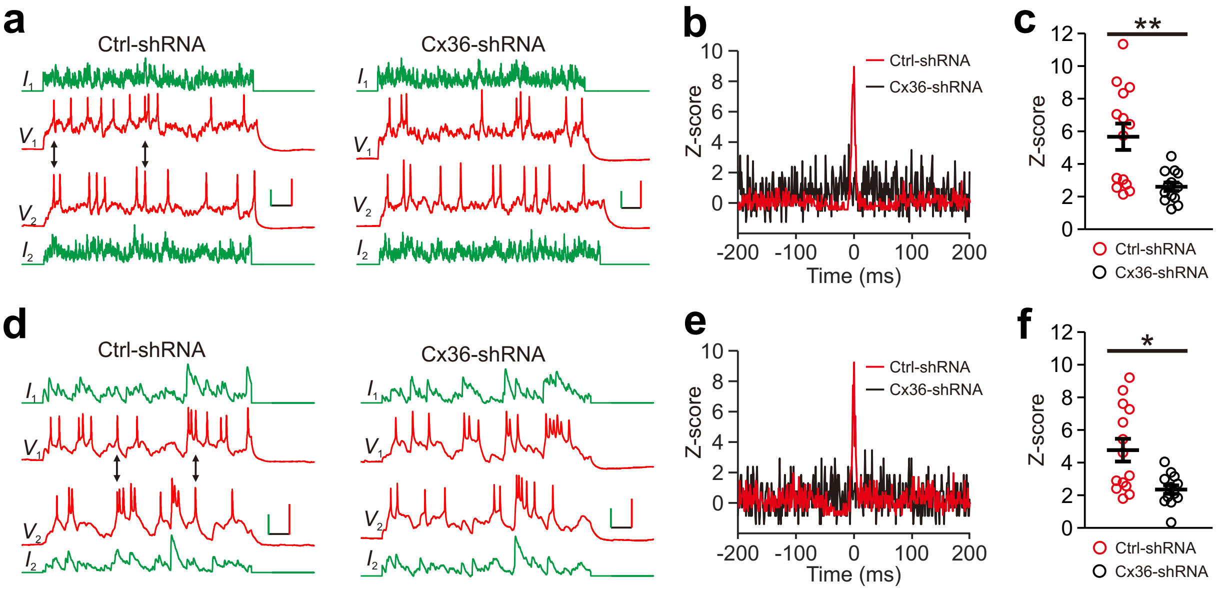


**Supplementary Figure 13. Electrical coupling is required for the synchronous firing of layer 1 interneurons (related to Fig. 10).** We injected two Ctrl-shRNA-expressed or Cx36-shRNA-expressed layer 1 interneurons with the uncorrelated simulated neuronal activity (**a-c**) or uncorrelated native neuronal activity (**d-f**) at P6-9, and then analyzed the cross-correlogram of firing induced in these layer 1 interneurons. (**a, d**) Sample traces of voltage changes in layer 1 interneurons in Ctrl-shRNA group and Cx36-shRNA group. Two-way arrows indicate the spikes that occur in both neurons within a 1 ms window. Scale bars, 100 ms (black horizontal), 100 pA (green vertical), 40 mV (red vertical). (**b, e**) Normalized cross-correlogram (Z- score) analysis. The bin size is 1 ms. Note that the firing frequency is significantly increased near 0 ms for Ctrl-shRNA pairs (red) but not for Cx36-shRNA pairs (black), indicating synchronous firing. (**c, f**) The scatter plots of Z-scores of Ctrl-shRNA/Cx36-shRNA pairs. Note that the average Z-scores of Ctrl-shRNA pairs were significantly higher than that of Cx36-shRNA pairs in response to uncorrelated simulated neuronal activity (5.67 ± 0.81 for Ctrl-shRNA pairs, n = 14; 2.60 ± 0.25 for Cx36-shRNA pairs, n = 13, $P < 0.01$) (**c**), or uncorrelated native neuronal activity (4.77 ± 0.69 for Ctrl-shRNA pairs, n = 14; 2.35 ± 0.26 for Cx36-shRNA pairs, n = 13, $P < 0.05$) (**f**). *$P$<0.05, **$P$<0.01. Mann-Whitney rank sum test. Error bars represent mean ± s.e.m.

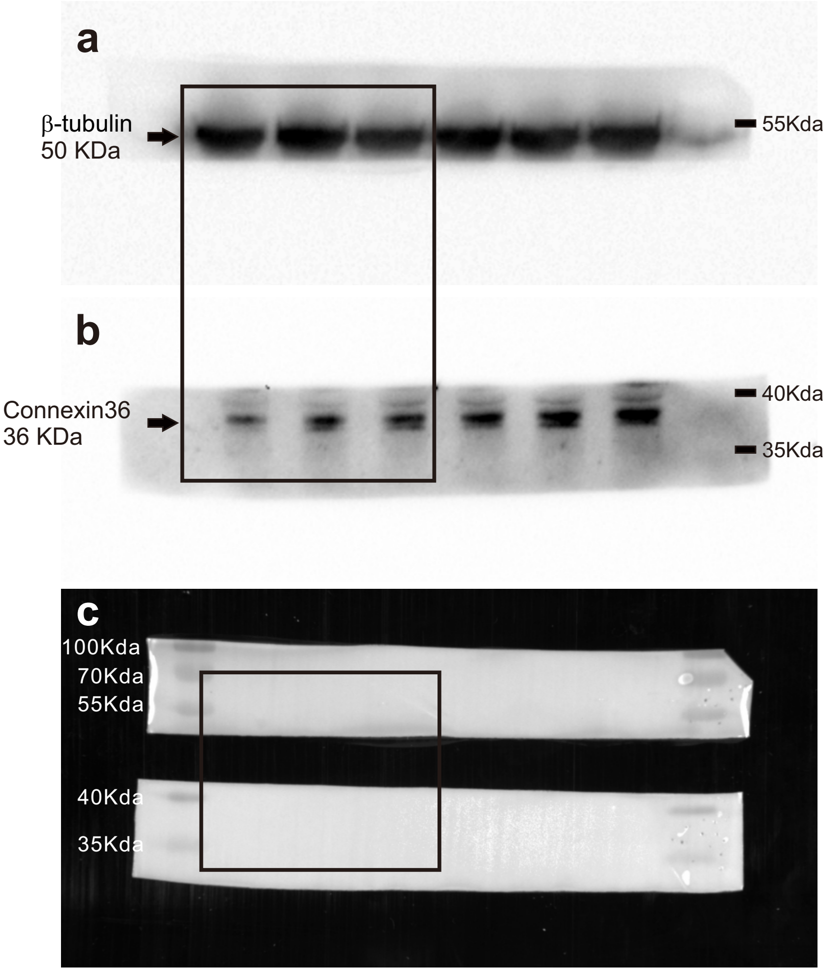


**Supplementary Figure 14. Uncropped gel images for Figure 6b.**

| | The postnatal stage of patch clamp recording | | | | | | | | | |
|---|---|---|---|---|---|---|---|---|---|---|
| | P1-2 | P3-4 | P5-6 | P7-8 | P9-10 | P11-12 | P13-14 | P15-16 | P17-18 | >P19 |
| Electrical connections | 0 | 0 | 6 (13.6%) | 26 (18.8%) | 57 (27.3%) | 68 (29.6%) | 20 (24.4%) | 39 (34.2%) | 14 (42.4%) | 35 (47.3%) |
| Unidirectional chemical connections | 0 | 0 | 7 (15.9%) | 33 (23.9%) | 61 (29.2%) | 62 (26.9%) | 28 (34.1%) | 32 (28.1%) | 8 (24.2%) | 17 (23.0%) |
| Bidirectional GABAergic connections | 0 | 0 | 1 (2.3%) | 19 (13.8%) | 23 (11.0%) | 38 (16.5%) | 15 (18.3%) | 12 (10.5%) | 3 (9.1%) | 14 (18.9%) |
| Total recording numbers | 9 | 14 | 44 | 138 | 209 | 230 | 82 | 114 | 33 | 74 |

**Supplementary Table 1.** Summary of proportion of electrical connections, and unidirectional and bidirectional chemical connections between layer 1 interneurons at different postnatal stages (related to Fig. 1g).

| ` | Ctrl-shRNA | | Cx36-shRNA | |
|---|---|---|---|---|
| | Amplitude (pA) | Frequency (Hz) | Amplitude (pA) | Frequency (Hz) |
| mEPSCs | 7.56 ± 0.64 | 1.45 ± 0.2 | 12.01 ± 1.91 | 4.02 ± 0.36 |
| mIPSCs | 14.55 ± 1 | 2.03 ± 0.15 | 12.54 ± 2.07 | 2.13 ± 0.46 |

Data are mean ± SEM.

**Supplementary Table 2.** Summary of the peak amplitude and frequency of mPSCs in Ctrl-shRNA and Cx36-shRNA groups (related to Fig. 9b-i).
